# *MIR937* amplification potentiates ovarian cancer progression by attenuating FBXO16 inhibition on ULK1-mediated autophagy

**DOI:** 10.1038/s41419-024-07120-8

**Published:** 2024-10-09

**Authors:** Zhen Zhang, Xinkui Liu, Chu Chu, Yingjie Zhang, Wei Li, Xiaoyan Yu, Qiaoqiao Han, Haoyu Sun, Yunhong Zhang, Xiaoxiao Zhu, Liang Chen, Ran Wei, Nannan Fan, Miaomiao Zhou, Xia Li

**Affiliations:** 1 Innovative Institute of Chinese Medicine and Pharmacy, Shandong University of Traditional Chinese Medicine, Jinan, China; 2 Key Laboratory of Traditional Chinese Medicine Classical Theory, Ministry of Education, Shandong University of Traditional Chinese Medicine, Jinan, China; 3 Shanghai Institute of Immunology, State Key Laboratory of Systems Medicine for Cancer, Shanghai Jiao Tong University School of Medicine, Shanghai, China; 4 Department of Gynecologic Oncology, Shandong Cancer Hospital and Institute, Shandong First Medical University and Shandong Academy of Medical Sciences, Jinan, China; 5 School of Clinical and Basic Medical Sciences, Shandong First Medical University and Shandong Academy of Medical Sciences, Jinan, Shandong China

**Keywords:** Ovarian cancer, Oncogenes

## Abstract

High-grade serous ovarian carcinoma (HGSOC) is one of the most lethal gynecological cancer. Genetic studies have revealed gene copy number alterations (CNAs) frequently occurred in HGSOC pathogenesis, however the function and mechanism of CNAs for microRNAs are still not fully understood. Here, we show the dependence on gene copy number amplification of *MIR937* that enhances cell autophagy and dictates HGSOC proliferative activity. Data mining of TCGA database revealed *MIR937* amplification is correlated with increased *MIR937* expression and cell proliferation of HGSOC. Deletion of *MIR937* in HGSOC cells led to impaired autophagy and retarded cell proliferation, and the extent for its inhibitory effects scaled with the degree of *MIR937* copy loss. Rescue assay confirmed miR-937-5p, a mature product of *MIR937*, was sufficient to restore its oncogenic function. Mechanistically, *MIR937* amplification raised the expression of miR-937-5p, enhanced its binding to 3′ UTR of *FBXO16* transcript, and thereby restricting FBXO16 degradative effects on ULK1. Our results demonstrate that *MIR937* amplification augments cell autophagy and proliferation, and suggest an alternative strategy of *MIR937*/FBXO16/ULK1 targeting for HGSOC treatment.

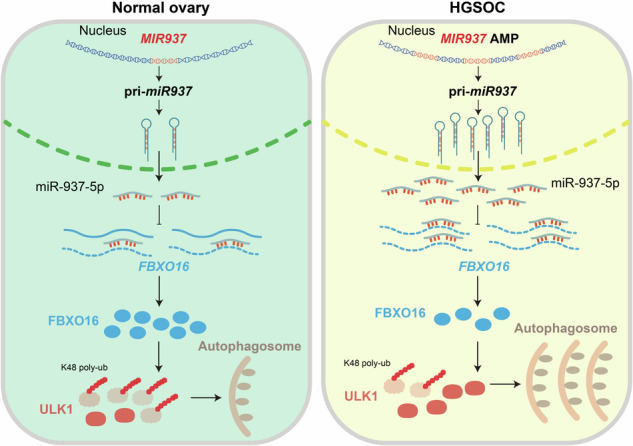

## Introduction

Ovarian cancer (OV) is a malignant tumor that poses serious threats to women’s health [1]. Among various histotypes of OV, high-grade serous ovarian cancer (HGSOC) is the most prevalent, accounting for 60–80% of new cases each year [2, 3]. It is estimated in clinic that more than 75% of HGSOC patients are diagnosed at advanced stages [4], which makes it most deadly and tough to be interfered with. So, the 5-year survival rate for HGSOC is less than 30% and has not been significantly improved over the last three decades for lack of early diagnostic markers and efficient therapeutic targets [5]. The need for more rigorous study has been strengthened to enhance our understanding of HGSOC tumor initiation and progression to provide with novel intervening strategies.

HGSOC patients are characterized by high frequency of somatic copy number alterations (CNAs), including deletion of tumor suppressors genes and amplification of oncogenes [6]. CNAs in most cases are typically correlated with changes of gene expression and protein abundances [7–9]. Increasing evidences have showed that gene amplification always causes proliferative phenotypes, attributing to enhanced expression of specific genes or the cumulative effects of gene collections [10]. In HGSOC, gain of function for gene amplification in tumorigenesis has been extensively studied. For instance, OV cells with genomic amplified URI relied on enhanced URI protein to maintain its survival [11]. USP13 amplification and over-expression reprogramed cancer cell metabolism, and aggravated tumor progression of HGSOC [12]. In addition, amplified RAD21 strengthened its interaction with YAP/TEAD4 to suppress interferon signaling and facilitated immune evasion of HGSOC cells [13]. So, researchers have been focusing on revealing the precision medical targets for related genomic alterations in clinic. HER2 antibody (trastuzumab), has got its way on the therapy of HER2 amplified breast cancer [14]. Although not for HGSOC, such success brings an inspiring possibility that targeting of amplified tumor drivers holds great potential for cancer treatment. Not only for protein-coding genes, CNAs of small non-coding microRNAs (miRNAs) also represent a type of genomic aberrations with cancer driving capabilities [15]. However, lack interpretation of the underlying mechanism for miRNA amplification in carcinogenesis has seriously limited the progress for HGSOC treatment.

miRNAs are composed of 19–24 nucleotides in length, and have profound biological functions in the aspects of cell survival, proliferation, and differentiation primarily by complementary binding of mRNA targets [16]. Functional studies have confirmed that miRNA can also act as tumor suppressors or oncogenes, and genomic aberrations of miRNAs can lead to miRNAs dys-regulation and constantly associate with cancer progression [17, 18]. The notion has been validated in HGSOC that copy number gain of miR-569 increased its expression and boosted tumor survival and proliferation by attenuating TP53INP1 transcription [19]. miRNA intervention by mimics or inhibitors (with anti-miR function) has shown great potential in clinic treatment for cancer and related diseases. For example, a mimic of tumor suppressor miR-16 (MesomiR-1), and an anti-miR targeting miR-155 (MRG-106) both reached phase I trial for treating malignant pleural mesothelioma and cutaneous T cell lymphoma, respectively [20, 21]. Given the prevalence of miRNA CNAs in cancers [22, 23] and the feasibility of manipulating miRNA expression in recipient, it encourages us to unravel the functional mechanism for amplified miRs in HGSOC to provide with prospective therapeutic targets.

In this study, we screened the amplified miRs in the frequently amplified locus of 8q24.3 in HGSOC patients by TCGA database. We found *MIR937* amplification can promote proliferation of HGSOC cells. Mechanistically, *MIR937* amplification increased miR-937-5p expression, reduced FBXO16 and its degradative effects on ULK1 protein, and thus enhanced autophagy. Importantly, our study revealed the regulatory axis for HGSOC progression and shed light on the novel combinational therapeutic targets for the patients.

## Materials and methods

### Cell culture and tissue samples

HEK293T, OVCAR3, A2780, TOV21G, and SKOV3 cells were obtained from Procell Life Science & Technology Co., Ltd (Wuhan, China). HEK293T cells were maintained in low-glucose Dulbecco’s Modified Eagle Medium (DMEM) with 10% fetal bovine serum at 37 °C in 5% CO_2_. OVCAR3, A2780, TOV21G, and SKOV3 cell lines were grown in RPMI medium with 10% fetal bovine serum at 37 °C in 5% CO_2_. All the cell lines were confirmed to have no contamination of mycoplasma. Frozen tissue specimens of normal ovary and ovary with epithelial malignant tumor were received from Cancer Hospital Affiliated to Shandong First Medical University. And the informed consent was obtained from all subjects.

### Plasmids, antibodies, and reagents

Human ULK1, FBXO16, CUL1, SKP1, and RBX1 genes were amplified and subcloned into the pcDNA3.1 vector with Flag or Myc tag. HA-tagged WT, K48-, and K63- Ub encoding plasmids were subcloned into the pcDNA3.1 vector. Antibodies against ULK1 (catalog 8054S), p62 (catalog 23214S), LC3A/B (catalog 4108S), CUL1 (catalog 4995S), ATG7 (catalog 8558S), ATG13 (catalog 13273S), Beclin-1 (catalog 3495S), Flag (catalog 14793S), Myc (catalog 2278S), and HA (catalog 3724S) were purchased from Cell Signaling Technology. Antibody against GAPDH (catalog ab181602) was obtained from Abcam. Anti-FBXO16 antibody (catalog LS-C82721) was purchased from LSBio. Cell counting kit-8 (CCK-8) (catalog C0038), d-luciferin potassium salt (catalog ST196), and crystal violet staining solution (catalog C0121) were purchased from Beyotime Biotechnology. CHX was purchased from MedChemExpress (catalog HY-12320). Cell-Light EdU Apollo567 In Vitro Kit (catalog C10310-1) was purchased from Ribobio.

### Generation of *MIR937* KO cell lines

For *MIR937* knockout, pLentiCRISPR v2 (Addgene, catalog 52961) was digested with KpnI (NEB, catalog R3142V) and EcoRI (NEB, catalog R0101V). Two sgRNAs flanking *MIR937* genomic locus were designed to delete *MIR937* encoding sequence. The sequence for the 1st sgRNA is TGCCCCCGGTGAGTCAGGGT, and the sequence for the 2nd sgRNA is GTTCCCGAGCTCCTGCAGGT, which were driven by U6 or H1 promoter, respectively. The encoding cassette for U6 promoter-sgRNA1-H1-promoter-sgRNA2 was synthesized, amplified, and ligated into pLentiCRISPR v2. The constructed plasmid, pMD2.G (addgene, catalog 12259) and psPAX2 (addgene, catalog 12260) were co-transfected into HEK293T cells for virus package. OVCAR3 and A2780 cells were infected, selected with puromycin (MCE, catalog HY-B1743A), and single cell was screened for *MIR937* knockout validation.

### Genomic DNA isolation, PCR, and copy number validation

Total genomic DNA was extracted by a DNA isolation Kit (Vazyme, DC112-01) for both in vitro cultured OV cell lines and human ovary tissue. For *MIR937* knockout analysis, genomic DNA of cultured OV cell lines was used as template for PCR amplification and sequencing with the following pair of primers: F: GTGGGGGCGTATAGTCTCTTG, R: GCATCGGTTAGTGTGCCTG. For clinical samples, FAM-labeled probe and the amplifying primers for genomic *MIR937* sequence were designed, VIC-labelled Rnase-P probe and primers were used to quantify the copy number of the reference gene, and the genomic DNA from TOV21G cells was used as diploid control. TaqMan copy number assay was performed with TaqMan Fast Advanced Master Mix (Thermo^TM^ Fisher Scientific, catalog 4444554). The results were collected on Quantstudio 1 Plus, and analyzed by CopyCaller Software (Thermo^TM^ Fisher Scientific).

### Generation of stable cell lines

FBXO16 coding sequence was sub-cloned into pCDH-GFP vector with Flag tag. ShRNA oligonucleotides targeting *FBXO16* were annealed into pLKO.1-EGFP vector. The sequences for shRNA are listed as follows: FBXO16-shRNA1: 5′-GCTATTGAATGACCGGGTA-3′; FBXO16-shRNA2: 5′-CAAGCTTCCAAGGGTGTTA-3′. The related plasmids were packaged into lentivirus with psPAX and pMD2.G plasmids for OVCAR3 infection, and the infected OVCAR3 cells were selected with puromycin at 1 μg/ml.

### MiRNA, siRNA transfection

miR-937-5p/3p mimics, mimics negative control (NC), inhibitors, or inhibitor NC oligonucleotides were transfected into OVCAR3, A2780, TOV21G, and SKOV3 cells, by HiperFect Transfection Reagent (Qiagen, catalog 301704) according to manufacturer’s instructions. For transient silencing of FBXO16, duplexs of siRNA were transfected into OV cells with Lipofectamine^TM^ RNAiMAX reagent (Thermo Fisher Scientific, catalog 13778075) according to the protocol. Target sequences used for FBXO16 knockdown were purchased from GenePharma. All the sequences used are available in Supplementary Table 1.

### RNA isolation and quantitative RT-PCR

Total RNA from tissues or cultured cells were isolated by FastPure Cell/Tissue Total RNA Isolation Kit (Vazyme, catalog RC101-01). RNA was reversed transcribed with the PrimeScript RT reagent Kit for mRNA (Toyobo, catalog RR037A) or with the miRNA 1st Strand cDNA Synthesis Kit (Vazyme catalog MR101) for microRNA following the manufacturers’ instructions. qRT-PCR assay was performed by using SYBR green (CWBIO, catalog CW2601H) on a Quantstudio 1 Plus instrument. Relative RNA expression was quantification via the comparative 2^−ΔΔCt^ method. The RT-qPCR primer sequences for relevant genes were listed in Supplementary Table 2. Primers for miRNAs detection were purchased from GenePharma, and U6 was used as control for microRNA expressional normalization.

### Cell proliferation and colony formation assay

OVCAR3, A2780, and TOV21G cells were seeded in 96-well dishes at 2000 cells per well, and SKOV3 cells were seeded with 1500 cells/well. Cell Counting Kit-8 (CCK-8) kit (Beyotime, catalog C0038) was used to determine cell count and viability, and the absorbance was measured at 450 nm by a spectro-photometric reader (MD, Spectramax 190). For colony formation assay, *MIR937* knockout OVCAR3 and A2780 cells were seeded into 6-well plates (600 cells/well), and cultured for 10 days. The colonies were fixed with 4% paraformaldehyde (PFA), and stained with 0.5% crystal violet. The number of colonies was counted by ImageJ software, and the data were represented as mean ± SD. Each experiment was repeated three times.

### 5-ethynyl-2′-deoxyuridine (EdU) incorporation assay

Transient transfected or *MIR937* knockout OVCAR3 and A2780 cells were seeded in 96-well plates with 1 × 10^4^ cells per well. Cell-Light EdU Apollo 567 In Vitro Imaging Kit (RiboBio, catalog C10310-1) were used to perform EdU assay according to the manufacturer’s instructions. In brief, after being incubated with 10 mM EdU for 2 h, the cells were fixed with 4% PFA, permeabilized in 0.5% Triton X-100, and labelled with fluorescent dye of Apollo 567. The nuclei were stained with DAPI (Solarbio, catalog C0065) for 15 min. Images were captured with a fluorescence microscope (Nikon, TS2R). EdU-positive cells were quantified with ImageJ software, and summarized as mean ± SD with at least three random fields.

### Dual-luciferase reporter assay

pGL3-3M-Luc vector was used to insert the 3′ un-translated region (UTR) of *FBXO16* transcript or its mutant 3′ UTR without miR-937-5p binding site. HEK293T cells were seeded in 24-well dishes one day before transfection. The WT or the mutant plasmids (with FBXO16 3′ UTR) were co-transfected with NC or miR-937-5p mimics. Forty-eight hours later, the cells were harvested and lysed, and Dual-Luciferase^®^ Reporter Assay System (Promega, catalog E1910) was used to measure the luciferase activity by a GloMax 20/20 Luminometer. The relative luciferase activity was normalized with Renilla activity. Three independent experiments were conducted.

### Immunoblotting and immunoprecipitation

Immunoblotting was performed as previously described [24]. In short, the cells were lysed with RIPA lysis buffer supplemented with PMSF (Solarbio, catalog P0100) and a protease inhibitor cocktail (Solarbio, catalog P1260). After centrifugation, the cell lysate was collected, boiled, and subjected to SDS-PAGE, and transferred to nitrocellulose (NC) filter membrane for immunoblotting analysis. As for immunoprecipitation analysis, IP buffer (50 mM Tris-HCl, 150 mM NaCl, 1.0% NP-40) with protease inhibitor cocktail was used to split the cells on ice for 15 min. The supernatant was collected and immunoprecipitated with agarose conjugated anti-Flag-Tag antibody (abmart, catalog M20018) overnight at 4 °C. The immunoprecipitates were washed with IP buffer three times, boiled in SDS sample buffer and analyzed by immunoblotting with indicated antibodies.

### In vitro binding and ubiquitination assays

ULK1 and FBXO16 recombinant proteins were prepared using a TNT Quick Coupled Transcription/Translation System (Promega). For the binding assay, ULK1 and FBXO16 were mixed together, followed by immunoprecipitation with the FBXO16 antibody and western blotting with the ULK1 antibody. The FBXO16-associated E3 complex was purified from the 293T cells transfected with Flag-CUL1/Myc-SKP1/Myc-RBX1 with the anti-Flag M2 affinity gel from Abmart. For in vitro ubiquitination assay, the purified E3 complex and FBXO16 were incubated in 30 μl reaction buffer containing 300 mM HEPES, pH 7.2, 50 mM MgCl_2_, and 2 mM dithiothreitol. Additional components included recombinant E1 activating enzyme (UBA1) and recombinant E2 (UBCH5). Purified recombinant monoubiquitins (WT, K48, K63) and 20 mM ATP were added to the reaction system, which was incubated for 1 h at 30 °C with shaking at 450 r.p.m. The reactions were terminated by adding Western blotting loading buffer and the products were resolved on SDS-PAGE.

### Immunohistochemistry (IHC)

The ovary tissues collected from control donors or the OV patients were formalin-fixed and paraffin-embedded (FFPE). The FFPE ovarian samples were sectioned and mounted on poly-lysine-coated slides. The sections were de-paraffinized and re-hydrated by xylene and gradient concentrations of ethanol, respectively. Then, antigen retrieval was fulfilled by 100 °C boiling of the section in 10 mM citrate buffer (Solarbio, catalog C1010) for 20 min. Then, a universal two-step detection kit (ZSGB-bio, catalog PV-9000) was used according to the manufacturer’s instructions for Immunohistochemistry (IHC) analysis. In detail, the endogenous peroxidase activity was blocked, and ULK1 antibody (Boster, catalog A00584-1) (1:250) diluted was incubated with the section overnight at 4 °C. The reaction enhancer reagent was used for 20 min before diaminobenzidine detection (ZSGB-bio, ZLI-9018).

### Confocal imaging

Autophagic flux was monitored by confocal microscopy. In brief, OVCAR3 cells with stable knockdown of FBXO16 were infected with GFP-RFP-LC3 adenovirus. Zeiss laser scanning confocal (LSM880) was used to measure autophagosomes (yellow puncta in merged images) and autolysosomes (red puncta in merged images) in normoxia and hypoxia conditions.

### Ovarian cancer xenograft mice model

Six-week-old female BALB/c athymic nude mice were obtained from the Huafukang BioScience company and bred under pathogen-free conditions. To establish subcutaneous xenograft models, OVCAR3 and A2780 cells (5 × 10^6^/mice) in 100 μl phosphate buffered saline were subcutaneously injected into the right flank of the nude mice. The size for xenograft tumor generated by *MIR937* wild type or genomic deleted OV cells was monitored every three days after its first appearance. For rescue assay, 4 × 10^6^ OVCAR3 or A2780 cells in PBS were subcutaneously injected, and agomiR-NC, agomiR-937-5p, and agomiR-937-3p (RiboBio) were directly injected into the implanted tumor at the dose of 1 nmol in 20 μl PBS every 4 days for five times. Tumor volume (V) was monitored by measuring the length (L) and width (W) with a Vernier caliper and calculated with formula *V* (cm^3^) = *W*^2^ × *L*/2. Finally, the mice were euthanized, and all tumors were excised, weighed. To establish intraperitoneal xenograft model, *MIR937*^+/+^, *MIR937*^+/−^, *MIR937*^−/−^ OV cells were transfected with pCDNA3.1-Luc plasmid, and screened with G418 for three days, and single cell was propagated with luciferase activity. 5 × 10^6^ luciferase labelled OVCAR3 or A2780 cells were injected intraperitoneally, and in vivo bioluminescence signals were collected by an animal imager (PerkinElmer, IVIS Lumina XRMS III) at day 7, 14, and 21 after inoculation by intraperitoneal injection of D-luciferin (150 mg/kg) (Beyotime, catalog ST196). All animal experiments were carried out in accordance with the Guide for the Care and Utilization of Laboratory Animals (Shandong University of Traditional Chinese Medicine) and were approved by the Institutional Animal Care and Use Committee of Shandong University of Traditional Chinese Medicine.

### Quantitative proteomics analysis

Proteomics analysis was executed by Jingjie PTM BioLabs (Hangzhou, China). In brief, 4D Label free proteomics analysis was conducted following the standard procedures: protein preparation, trypsin digestion, HPLC fractionation, LC–MS/MS analysis, and bioinformatics analysis.

### Statistics

All experiments were repeated at least three times, unless otherwise stated. Values are presented as the mean ± SD. Statistical differences between two groups was assessed by two-tailed Student’s *t*-test. While for multiple groups comparisons, statistical significance was evaluated by one-way analysis of variance (ANOVA) using GraphPad Prism 9.0 software. For correlation analysis, Pearson’s correlation was used for calculating *r* and *p*. *P* ≤ 0.05 was considered statistically significant.

## Results

### *MIR937* is amplified in HGSOC patients and correlates with increased expression of *MIR937*

**Fig. 1:**
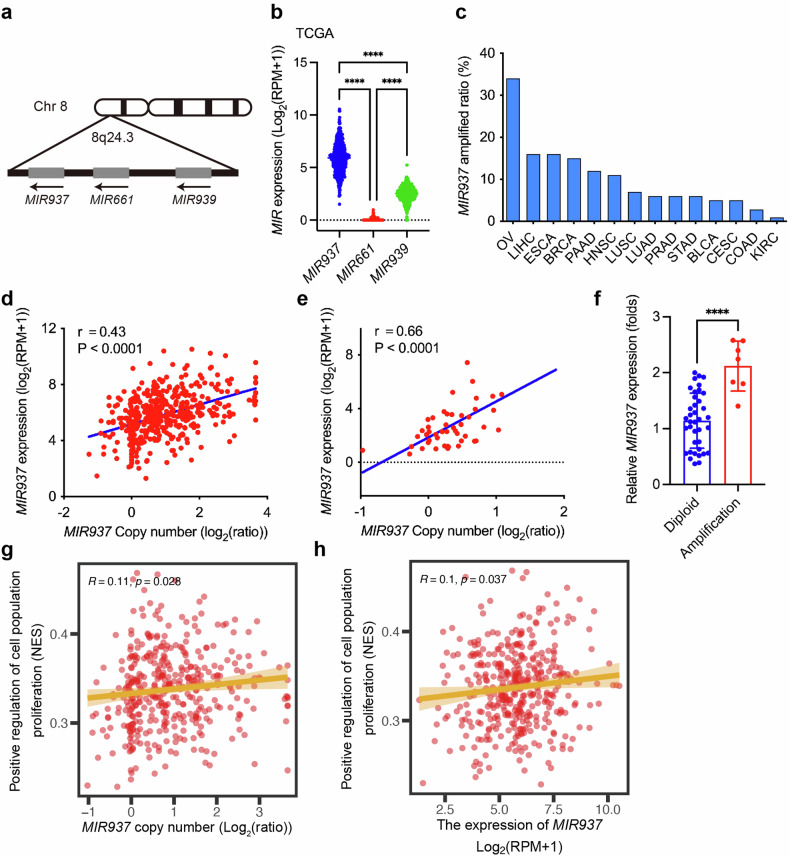
*MIR937* is amplified in HGSOC patients and correlates with increased expression of *MIR937.* **a** Schematic diagram of three miRs in the genome locus of 8q24.3. **b** The expressional data of three miRs were retrieved from cBioportal tools for TCGA database analysis. **c** Genomic amplification of *MIR937* across human cancers retrieved from cBioportal tools for TCGA databases analysis. The correlations between *MIR937* copy number and its expression were performed by the data from TCGA database (**d**) or CCLE OV cancer cell lines (**e**). **f** *MIR937* expression was quantified in HGSOC patients and the comparison between *MIR937* diploid and amplified patients was performed. **g** Pearson correlation between the *MIR937* copy number and the proliferation score calculated by ssGSEA. **h** Pearson correlation between the *MIR937* mRNA expression and the proliferation score calculated by ssGSEA. The gene set named “positive regulation of cell population proliferation” was obtained from the Gene Ontology (GO) knowledgebase (https://www.geneontology.org/). ssGSEA: single sample gene set enrichment analysis. Statistical analysis was performed using one-way ANOVA in (**b**), and two-tailed Student’s *t*-test in (**f**); Pearson correlation coefficients (R) were employed to evaluate the correlations between two continuous variables in (**d**, **e**, **g**, **h**). ****P < 0.001.

Somatic gene CNAs frequently occurred in the whole genome of HGSOC patients, which indicated that CNA genes might contribute to the pathogenesis of HGSOC. Genome-wide association study has been conducted and revealed that the genomic fragment of 8q24 is a susceptible locus for HGSOC patients [25]. Further, in this locus, 8q24.3 was one of the most significantly amplified fragments across the genome, which comprised 75 typical genes [26]. However, the large proportion of the genes located has not been functionally clarified in HGSOC. microRNA (miR) is small and short in genome sequence, and what interests us most is how the genomic alterations of such little fragments affects the progression of HGSOC. Impressively, there are three miRs located in 8q24.3 loci, *MIR937*, *MIR939*, and *MIR661* (Fig. 1a). Firstly, we checked the absolute expressional level for the three miRs in the HGSOC patients from The Cancer Genome Atlas (TCGA) database, and found the expression of *MIR937* is relatively high. At the same time, the expression of MIR939 and MIR661 is quite low (Fig.1b). We also examined the expression for these miRs in all OV cell lines from Cancer Cell Line Encyclopedia (CCLE) database. Consistent results were obtained (Supplementary Fig. 1a). So, we chose *MIR937* for further study. We systematically screened the amplification frequency of *MIR937* across different cancer types by TCGA database, and discovered *MIR937* amplified with the highest frequency in patients with HGSOC (Fig. 1c).

To further demonstrate the clinical significance of *MIR937*, we firstly conducted a correlation analysis between DNA copy number and expression of *MIR937* in HGSOC patients and cancer cell lines, and it was revealed that RNA level of *MIR937* positively correlated with its DNA copy number (Fig. 1d, e). In addition, the positive correlation was also observed in all cancer cells in CCLE (Supplementary Fig. 1b). Meanwhile, If the patients or OV cells lines were grouped according to CNA status of *MIR937*, it can also be found that the abundance of *MIR937* transcript elevated significantly upon its amplification (Supplementary Fig. 1c, d). To verify the correlation, we collected HGSOC patient samples, and by Taqman qPCR analysis, 7 out of 45 samples were validated with *MIR937* amplification, which showed significant higher expression of *MIR937* (Fig. 1f). The data indicated *MIR937* upregulation is a major consequence of *MIR937* amplification. Moreover, we performed gene ontology (GO) enrichment analysis by TCGA database, and discovered that *MIR937* copy number and expression are positively correlated with GO terms of “positive regulation of cell population proliferation” (Fig. 1g, h) and “negative regulation of programmed cell death” (Supplementary Fig. 1e, f). Collectively, our results implied that miR937 genomic amplification accounts much for its upregulation, and thus may aggravates its pathological effects in HGSOC occurrence.

### *MIR937* deficiency attenuates proliferation of ovarian cancer cells

**Fig. 2:**
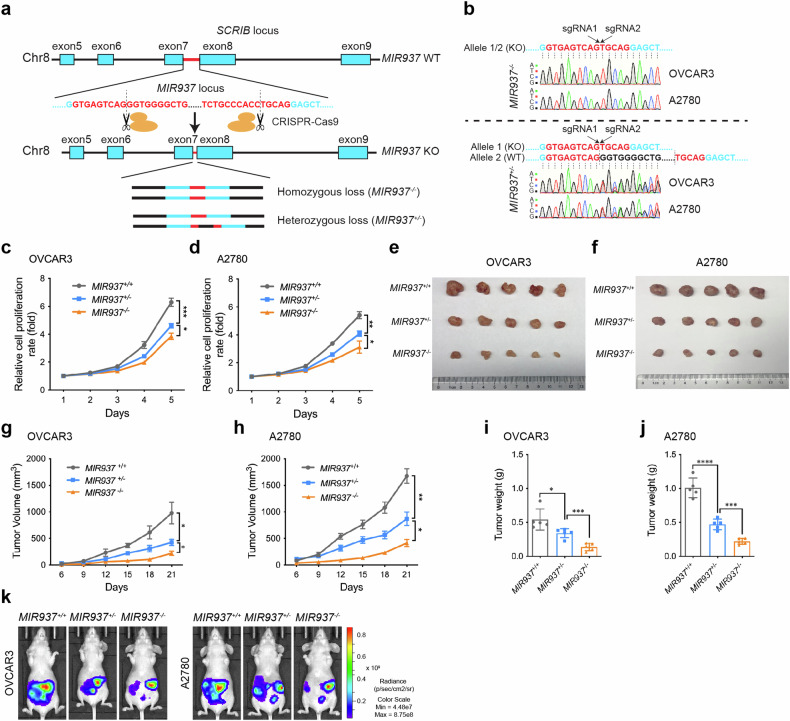
MIR937 deficiency attenuates proliferation of ovarian cancer cells. **a** CRISPR-Cas9 based knockout strategy for *MIR937* in OVCAR3 and A2780 cells. **b** Sanger sequencing for determined the genotype of *MIR937*^+/−^ and *MIR937*^−/−^ OVCAR3 and A2780 cells. Growth curve of *MIR937*^+/+^, *MIR937*^*+/*−^, and *MIR937*^*−/−*^ cells for OVCAR3 (**c**) and A2780 (**d**). The cells were seeded in 96-well plates with the initial number of 2000, and the growth status was continuously monitor for 5 days. Xenograft tumors derived from *MIR937*^+/+^, *MIR937*^*+/−*^, and *MIR937*^*−/−*^ OVCAR3 cells (**e**), and A2780 cells (**f**) were displayed. 5 × 10^6^ for each cell were s.c. injected into the right flank of nude mice (*n* = 5). The mice were sacrificed three weeks later after the inoculation. Growth curve for the xenograft tumors for OVCAR3 (**g**) and A2780 (**h**) cells with the indicated *MIR937* gene loss. The volume of tumors was measured every three days after their appearances (day 6). **i**, **j** Tumor weights were measured after mice were sacrificed. **k** Representative bioluminescence images of mice intraperitoneal injected with luciferase-expressing OVCAR3 and A2780 cells (*MIR937*^+/+^, *MIR937*^*+/−*^, and *MIR937*^*−/−*^) were collected at day 12 after inoculation. Data are shown as the mean ± SD. Similar results were obtained in three independent experiments. Statistical analysis was performed using one-way ANOVA in (**c**, **d**, **g**–**j**); *P < 0.05; **P < 0.01; ***P < 0.001; ****P < 0.0001.

To investigate the potential effect of *MIR937* on OV progression, we designed CRISPR-Cas9-based knockout strategy for *MIR937* in OVCAR3 and A2780 cells (Fig. 2a). In detail, two sgRNAs were used flanking the *MIR937* genome locus without affecting the adjacent exons for *SCRIB* encoding. Single cells with *MIR937* knockout were screened, validated by genomic DNA PCR amplification and Sanger sequencing. Homozygous and heterozygous loss of *MIR937* cells (*MIR937*^−/−^, *MIR937*^+/−^) were selected (Fig. 2b). We firstly monitored the genomic loss of *MIR937* on OV proliferation by CCK8 assay in vitro, and realized that *MIR937*^+/−^ grew slower than *MIR937*^+/+^ cells, while *MIR937*^−/−^ extended the inhibitory effect for both OVCAR3 and A2780 cells (Fig. 2c, d). In addition, colony formation assay revealed the capacity of the cells to form colonies declined gradually with *MIR937* gene loss (Supplementary Fig. 2a, c). The statistical results also demonstrated the significant loss for the colony formation ability with *MIR937* knockout (Supplementary Fig. 2b, d). EdU incorporation assay was also performed, which further proved that *MIR937* deficiency inhibited the proliferative features for both OVCAR3 and A2780 cells (Supplementary Fig. 2e). The statistical data showed EdU positive cells were reduced by heterozygous loss of *MIR937* to 78% or 70%, but reduced by homozygous loss of *MIR937* to 42% or 44% respectively for OVCAR3 and A2780 cells (Supplementary Fig. 2f, g). The in vitro data highly indicated that deficiency degree of *MIR937* correlated with inhibitory extent for proliferation of OV cells.

To further validate the function of *MIR937*, we firstly subcutaneously injected *MIR937*^+/+^, *MIR937*^+/−^, *MIR937*^−/−^ cells to the right flank of the nude mice to establish xenograft models. The mice were sacrificed when the tumor grew to the appropriate size to visualize the differences of tumor growth for different groups (Fig. 2e, f). By monitoring the growth rate, and the weight for the xenograft tumor, we consistently found *MIR937*^*−/−*^ cells exhibited more restricted cell growth than *MIR937*^+/−^ cells (Fig. 2g–j). Next, we generated luciferase labelled OVCAR3 and A2780 cells with *MIR937* deficiency, and injected these cells intraperitoneally to mimic native environment for OV tumor growth. The in vivo luciferase signal captured consistently denoted that *MIR937* deficiency could severely suppress proliferation of OV cells (Fig. 2k). In general, these data indicated that *MIR937* might play an oncogenic role in OV development.

### miR-937-5p accounts for MIR937 loss of function in ovarian cancer cells

**Fig. 3:**
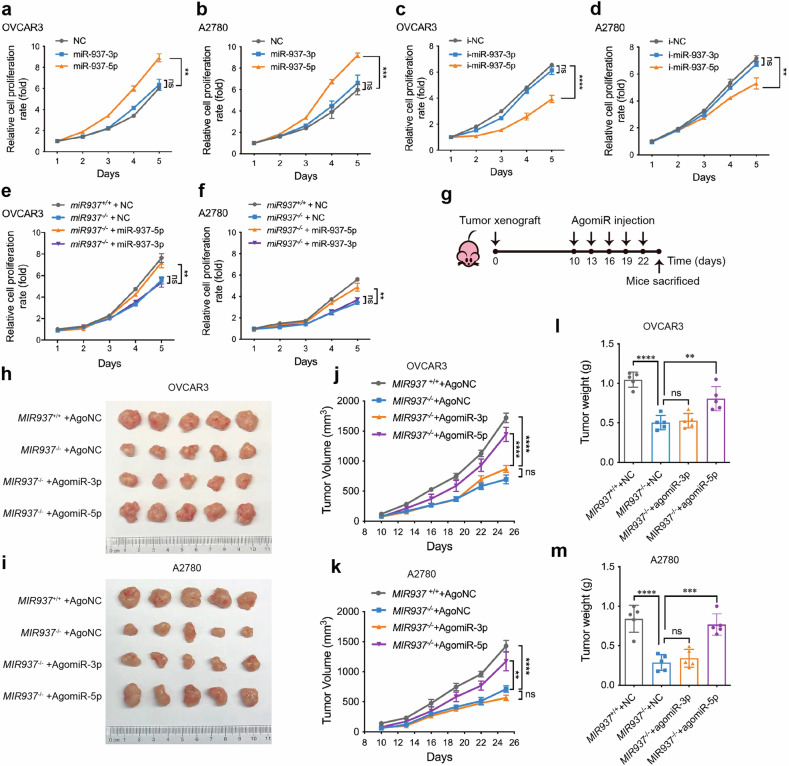
miR-937-5p accounts for MIR937 loss of function in ovarian cancer cells. Growth curves for OVCAR3 (**a**) and A2780 (**b**) cells were monitored by CCK8 analysis upon miR-937-5p or 3p mimics transfection. **c**, **d** Growth curves for OVCAR3 and A2780 cells were detected after transfection of miR-937-5p or 3p inhibitors. Growth curves for OVCAR3 (**e**) and A2780 (**f**) comparison of *MIR937*^+/+^ cells with *MIR937*^−/−^ cells, into which miR-937-5p or 3p mimics were transfected. *MIR937*^+/+^ cells mock transfected, while *MIR937*^−/−^ cells were transfected with miR-937-5p or 3p mimics (**g**) The graphical summary for agomiR injection into the xenograft tumors. Xenograft tumors derived from OVCAR3 (**h**) and A2780 (**i**) cells were displayed. *MIR937*^+/+^ tumors were mock treated with agomiR-NC, and for *MIR937*^−/−^ tumors, agomiR-937-5p and -937-3p were used to interfere with tumor growth. Growth curve for the xenograft tumors affected by agomiR-937-5p and -937-3p for OVCAR3 (**j**) and A2780 cells (**k**). Tumor weights for OVCAR3 (**l**) and A2780 (**m**) cells were measured at day 25 when the mice were sacrificed after five rounds of agomiR injection. Data are shown as the mean ± SD. Similar results were obtained in three independent experiments. Statistical analysis was performed using one-way ANOVA in (**a**–**f**, **j**–**m)**; **P < 0.01; ***P < 0.001; ****P < 0.0001.

*MIR937* transcribed as a precursor, and finally resulted in the formation of two mature products, miR-937-5p and miR-937-3p. Whether miR-937-5p or miR-937-3p is responsible for *MIR937* oncogenic role remains to be illustrated. So, firstly, mimics or inhibitors of the two miRs were used to over-express or repress miR-937-5p and miR-937-3p expression in OVCAR3 and A2780 cells (Supplementary Fig. 3a–d). By CCK8 analysis, we discovered overexpression of miR-937-5p significantly enhanced the proliferation rate for both cell lines, while miR-937-3p has no effect (Fig. 3a, b). Consistently, the inhibitors of miR-937-5p but not miR-937-3p could inhibit the proliferation of OVCAR3 and A2780 cells (Fig. 3c, d). In addition, miR-937-5p could also improve the proliferation ability of TOV21G and SKOV3 cells (Supplementary Fig. 3e, f). Meanwhile, EdU assay was also performed, and the results showed that miR-937-5p over-expression could significantly increase the proportion of EdU-positive cells (Supplementary Fig. 3g, h). It highly suggested that miR-937-5p was the major product responsible for *MIR937* oncogenic effects.

Then, we over-expressed miR-937-5p or miR-937-3p in *MIR937*^−/−^ cells to further investigate which product could rescue *MIR937* deficiency caused arrest of OV cell growth. We found that only miR-937-5p mimics transfection could rescue the growth ability of *MIR937*^−/−^ cells close to that of *MIR937*^+/+^ cells (Fig. 3e, f). Finally, we established subcutaneous xenograft tumors by *MIR937*^+/+^, and *MIR937*^−/−^ cells of OVCAR3 and A2780, into which agomiR-937-5p and agomiR-937-3p were injected every 3 days for five times (Fig. 3g). At day 25, the mice were sacrificed, and the xenograft tumors were measured in size and weight to confirm the effects for both miRs. When being injected with agomiR-937-5p, but not agomiR-937-3p, the size for *MIR937*^−/−^ xenografts increased significantly (Fig. 3h, i). Furthermore, agomiR-937-5p rescued the proliferation rate and the weight for the xenograft tumors of *MIR937*^−/−^ OVCAR3 and A2780 cells (Fig. 3j–m). Taken together, our data consolidated that miR-937-5p is the product of *MIR937* gene for its oncogenic roles.

### miR-937-5p directly targets the tumor suppressor gene of FBXO16

**Fig. 4:**
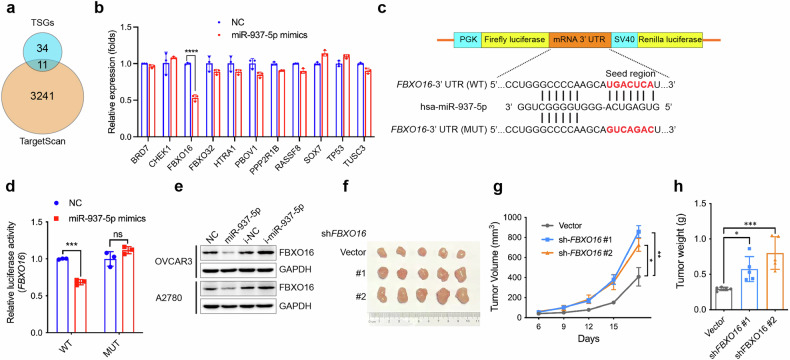
miR-937-5p directly targets the tumor suppressor gene of FBXO16. **a** Venn diagram showing the overlapping 11 potential targets by lists of TSGs and targets of miR-937-5p acquired from TargetScan database. **b** The expression of 11 predicted genes were detected by qPCR analysis upon miR-937-5p mimics transfection in OVCAR3 cells. **c** Potential miR-937-5p binding sequence in the 3′ UTR of FBXO16 mRNA predicted by TargetScan. The sequence of the mutant construct for luciferase reporter assay was also indicated. **d** Relative luciferase activity was monitored after the former described reporter plasmids co-transfected with miR-937-5p mimics in HEK293T cells. The firefly luciferase activity was normalized to renilla activity. **e** FBXO16 protein level was detected following transfection of miR-937-5p mimics or inhibitors in OVCAR3 and A2780 cells. GAPDH was used as a loading control. **f** Xenograft tumors derived from OVCAR3 cells were displayed. Two cell lines of OVCAR3 with FBXO16 stable knockdown and one control cell line (vector) were s.c. injected into nude mice. **g** Growth curve for the xenograft tumors generated by OVCAR3 cells with stable knockdown of FBXO16. **h** Tumor weights were measured on day 21 after the mice being sacrificed. Data are shown as the mean ± SD. Similar results were obtained in three independent experiments. Statistical analysis was performed using two-tailed Student’s *t*-test in (**b**, **d**) and one-way ANOVA in (**g**, **h**); *P < 0.05; ***P < 0.001; ****P < 0.0001.

Since miR-937-5p is functional in promoting tumor growth, we hypothesized that miR-937-5p might target to some tumor suppressor genes (TSGs) in OV. To ensure accurate screening of miR-937-5p target genes, we conducted a detailed literature search using keywords of “HGSOC” and “tumor suppressor genes”. We manually curated 45 tumor suppressor genes, which had been verified by different research teams, for target screening. We also used the TargetScan database to get the list of potential miR-937-5p targets. When overlapping the genes from the two lists, eleven TSGs were revealed to be likely targeted by miR-937-5p (Fig. 4a). To verify whether the genes were truly targeted by miR-937-5p, we transfected miR-937-5p mimics in OVCAR3 cell, quantified the expression of the indicated genes, and found miR-937-5p could only reduce the expression of *FBXO16* (Fig. 4b). Conversely, deficiency of *MIR937* in the OVCAR3 cells greatly elevated mRNA level of *FBXO16* (Supplementary Fig. 4a). In addition, one binding site in the 3′ UTR of *FBXO16* was discovered, and luciferase reporter analysis displayed that miR-937-5p inhibited luciferase activity with WT 3′ UTR but did not affect that with mutant 3′ UTR (Fig. 4c, d). To directly test whether miR-937-5p binding of FBXO16 3′ UTR inhibited its protein translation, we transfected mimics and inhibitors and detected the influence of miR-937-5p on protein levels of FBXO16 in OVCAR3 and A2780 cells. It showed that miR-937-5p mimics remarkably reduced, while the inhibitors enhanced the expression of FBXO16 (Fig. 4e). Consistently, *MIR937* loss coupled with FBXO16 protein increase (Supplementary Fig. 4b). What’s more, we also submitted the samples of xenograft tumors for western blot analysis, and found that agomiR-937-5p could reduce FBXO16 protein levels that had been enhanced by *MIR937* loss (Supplementary Fig. 4c). Collectively, these data confirmed that FBXO16 is the bona fide target of miR-937-5p in OV cancer cells.

In addition, the tumor suppressive effects of FBXO16 for OV have been further verified in our model of study. We silenced FBXO16 expression by three small interfering RNAs (siRNAs) (Supplementary Fig. 4d) and detected the effect of FBXO16 on cell proliferation. By CCK8 analysis, we found that all the siRNAs targeting FBXO16 could significantly enhanced the proliferative activity for OVCAR3 cells (Supplementary Fig. 4e). Besides, two short hairpin RNAs (shRNAs) targeting FBXO16 were also constructed to generate stable cell lines with FBXO16 silencing (Supplementary Fig. 4f). It was also revealed that stable knockdown of FBXO16 could consistently improve the proliferation rate of OVCAR3 cells (Supplementary Fig. 4g). For in vivo assay, both shRNAs could remarkably increase the size, growth rate, and weight of tumors by the subcutaneous xenograft model (Fig. 4f–h). In summary, our data demonstrated that miR-937-5p targeted FBXO16, thus reduced its tumor suppressive effects and played oncogenic roles.

### FBXO16 negatively regulates autophagy in ovarian cancer

**Fig. 5:**
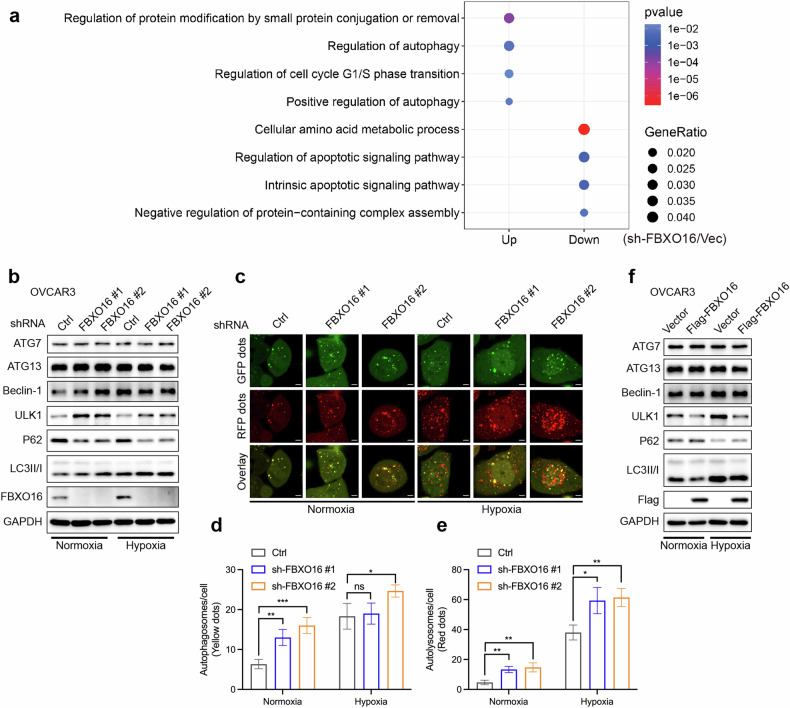
FBXO16 negatively regulates autophagy in ovarian cancer. **a** Gene ontology enrichment analysis of upregulated (Up) and downregulated (Down) gene sets in OVCAR3 cells with stable knockdown of FBXO16. **b** IB analysis for autophagy-related proteins affected by stable knockdown of FBXO16 in OVCAR3 cells under normal and hypoxia conditions. The cells were placed in the anaerobic incubator with 1% oxygen at 37 °C for 12 h. Similar results were obtained in three independent experiments. **c**–**e** OVCAR3 cells with stable knockdown of FBXO16 were infected with GFP-RFP-LC3 adenovirus for 48 h, then cells were placed in the anaerobic incubator with 1% oxygen at 37 °C for 12 h. Autophagic flux was monitored by confocal microscopy (**c**). The mean number of autophagosomes per cell (yellow puncta in merged images) (**d**) and autolysosomes (red puncta in merged images) (**e**) were counted. **f** IB analysis for autophagy-related proteins affected by over-expression of FBXO16 in OVCAR3 cells under normal and hypoxia conditions.

To demonstrate the molecular mechanism by which *MIR937*/*FBXO16* regulate OV proliferation, we firstly tested the influence of *MIR937* loss on hnRNPL, a formerly reported target of FBXO16. The results showed no dramatic changes of hnRNPL in *MIR937*^−/−^ compared with that of *MIR937*^+/+^ cells (Supplementary Fig. 5a). It indicated that *MIR937* might exert its oncogenic effects through a distinctive role of FBXO16. So, we sought to uncover novel targets of FBXO16 in OV. 4D label-free quantitative proteomic analysis was performed to screen the biological processes potentially affected in OVCAR3 cells with stable knockdown of FBXO16. GO enrichment analysis for differentially expressed proteins was performed and the results showed that cell cycle, and autophagy-related pathways were significantly upregulated upon FBXO16 silencing, whereas cell apoptosis and amino acid metabolic-related processes were downregulated (Fig. 5a). Since autophagy is an essential process that regulates cell proliferation, cell death, and cell metabolism, we asked whether FBXO16 affected the expression of key regulatory proteins for autophagy. By western blot analysis, we found FBXO16 knockdown increased the protein level of ULK1, but not ATG7, ATG13, or Beclin-1, and could simultaneously enhance the ratio of LC3II/I, but reduce p62 under both normal and hypoxic conditions (Fig. 5b). We further monitored autophagic flux by confocal microscopy in OVCAR3 cells infected with GFP-RFP-LC3 adenovirus. The results showed that both shRNA targeting FBXO16 could enhance basic and hypoxia-induced autophagy flux (Fig. 5c–e). Stable over-expression of FBXO16 displayed remarkable reduction of ULK1 protein, and suppression of cellular autophagy reflected by LC3II/I ratio and p62 (Fig. 5f). In addition, the result that FBXO16 only targeted ULK1 was further confirmed with transient knockdown of FBXO16 by three siRNA in both A2780 and TOV21G cells (Supplementary Fig. 5b, c). Furthermore, we assessed the impact of FBXO16 on the phosphorylation level of ULK1. The results indicated that the knockdown of FBXO16 significantly elevated ULK1 phosphorylation, whereas the over-expression of FBXO16 resulted in a reduction of ULK1 phosphorylation under both normal and hypoxic conditions (Supplementary Fig. 5d, e). Collectively, our findings suggest that FBXO16 modulates ULK1 activation by influencing its protein abundance, thereby regulating the initiation of autophagy.

### FBXO16 interacts with ULK1 and facilitates its K48-linked poly-ubiquitination

**Fig. 6:**
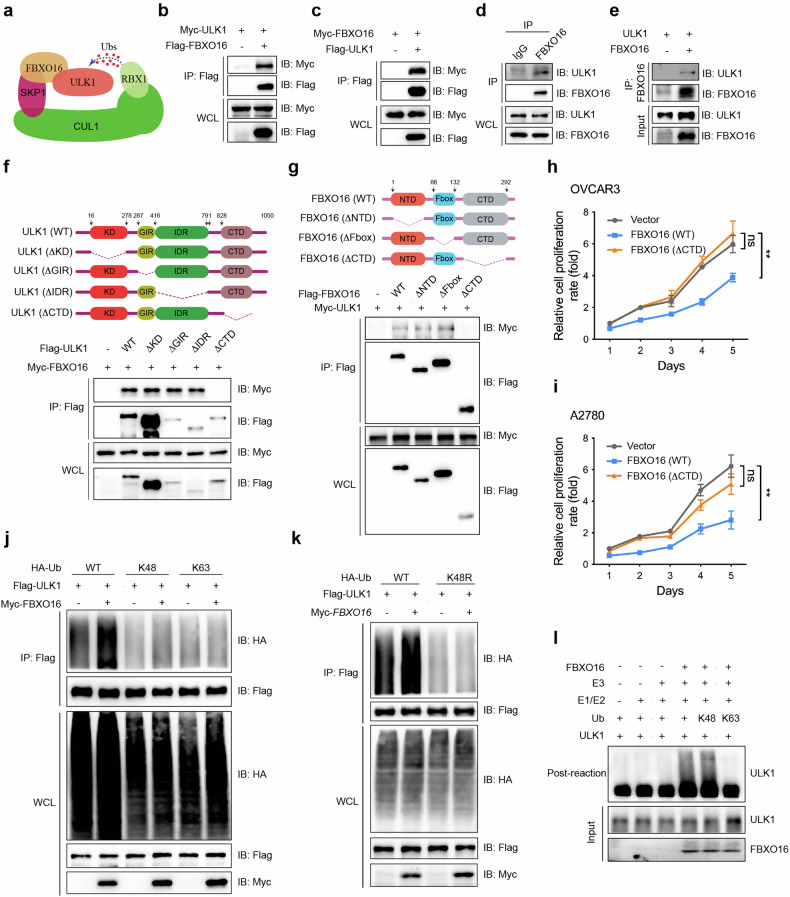
FBXO16 interacts with ULK1 and facilitates its K48 linked poly-ubiquitination. **a** Schematic presentation of predicted interaction between ULK1 and CUL1-SKP1-FBXO16 complex. Co-IP analysis with anti-Flag antibody to detect the interaction between Flag-FBXO16 and Myc-ULK1 (**b**), and Myc-FBXO16 and Flag-ULK1 (**c**) in HEK293T cells co-transfected with the indicated plasmids. **d** Lysates from OVCAR3 cells were subjected to immunoprecipitation analysis with anti-FBXO16 antibody, followed by immunoblotting analysis with anti-FBXO16 and anti-ULK1 antibodies, respectively. **e** Recombinant FBXO16 and ULK1 proteins were prepared in an in vitro transcription and translation system, immunoprecipitation analysis was performed using anti-FBXO16 antibody, immunoblot analysis was followed with anti-ULK1 antibody. Wild type (WT) and truncated mutant schematic structures of ULK1 (**f**) and FBXO16 (**g**) were presented (upper), and co-IP with Flag-antibody was conducted to visualize the interaction between FBXO16 with ULK1 truncations (lower), and ULK1 with FBXO16 truncated mutants (lower). OVCAR3 (**h**) and A2780 (**i**) cells were infected with lenti-virus to over-express FBXO16 (WT) and FBXO16 (∆CTD) proteins, and CCK8 assay was performed to detect the proliferation of both cells. Co-IP with anti-Flag antibody followed by immunoblot analysis (IB) to detect ULK1 ubiquitination affected by FBXO16 in HEK293T cells. WT, K48, and K63 mutant forms of HA-Ubs were used in (**j**), while WT and K48R mutant of HA-Ubs were used in (**k**). Similar results were obtained in three independent experiments. **l** Recombinant ULK1 and FBXO16 proteins were prepared in an in vitro transcription and translation system. In vitro ubiquitination assay was performed in the presence of E1, E2, FBXO16, ULK1, Ubs (WT, K48, K63), and immunoprecipitated CUL1-SKP1-RBX1 complex. The ubiquitination of ULK1 was examined by immunoblot analysis with anti-ULK1 antibody.

FBXO16 always acts as an adapter that recruits the substrate to SKP1-cullin 1 (CUL1)-RBX1 complex for its ubiquitination and subsequent degradation (Fig. 6a). So, we checked whether FBXO16 could interact with ULK1 to possibly affect its protein stability. By co-immunoprecipitation assay, we found ectopically expressed Flag-tagged FBXO16 strongly interacted with Myc-tagged ULK1 (Fig. 6b). Meanwhile, Flag-tagged ULK1 could also bind to Myc-tagged FBXO16 (Fig. 6c). We also detected the interaction between endogenous FBXO16 and ULK1 in OVCAR3 cells (Fig. 6d). To verify that FBXO16 associated with ULK1 directly, FBXO16 and ULK1 recombinant proteins were prepared and in vitro immunoprecipitation was performed. These results revealed that FBXO16 could form a complex with ULK1 directly (Fig. 6e). To determine the binding requirements for FBXO16-ULK1 complex, we generated three and four deletion constructs of FBXO16 and ULK1, respectively, and performed the co-IP assay. The results showed that the C-terminal domains (CTD) of FBXO16 and ULK1 are responsible for their interaction (Fig. 6f, g). Then, we transfected A2780 and OVCAR3 cells with plasmids encoding FBXO16 (WT) and FBXO16 (∆CTD) proteins, and by CCK8 analysis, we confirmed the necessity of CTD domain for FBXO16 inhibitory function on OV cell proliferation (Fig. 6h, i). We further detected ULK1 interaction with the key components of SCF complex by co-IP analysis, and unveiled that Flag-tagged ULK1 could bind to Myc-tagged CUL1, SKP1, and RBX1 (Supplementary Fig. 6a–c). Besides, knockdown of FBXO16 could significantly suppress the interaction of ULK1 and CUL1 or RBX1 (Supplementary Fig. 6d, e). This highly implied that ULK1 was recruited to SCF complex by FBXO16.

Further, we co-transfected HEK293T cells with Flag-ULK1, Myc-FBXO16, and HA-Ubs (WT, K48, and K63). Cell lysates were immunoprecipitated with Flag antibody, and poly-ubiquitinated ULK1 was detected by western blot. It was revealed FBXO16 could significantly increase K48, but not K63, linked poly-ubiquitination of ULK1 (Fig. 6j). Consistently, FBXO16 knockdown in OVCAR3 cells showed reduction of both WT and K48 linked poly-ubiquitination of ULK1 (Supplementary Fig. 6f). On the other hand, we transfected Flag-ULK1, Myc-FBXO16, and HA-Ubs (WT, K48R) into HEK293T cells, and the cell lysate were subjected to co-IP analysis. The results illustrated that lysine-48 (K-48) mutation to arginine (R) of Ub blocked FBXO16-mediated ubiquitination of ULK1, which further validated that FBXO16 facilitated K48-linked poly-ubiquitination of ULK1 (Fig. 6k). We also performed in vitro ubiquitination assay in the presence of recombinant FBXO16 and ULK1 proteins, and the results showed that FBXO16 is responsible for K48 poly-ubiquitination of ULK1 (Fig. 6l). To further demonstrate that FBXO16 facilitated ULK1 degradation via ubiquitination, we transiently knockdown FBXO16, and treated the cells with cycloheximide (CHX) to detect the stability of ULK1 protein affected by FBXO16. The data showed that FBXO16 knockdown increased ULK1 protein level and slowed down its degradation in both OVCAR3 (Supplementary Fig. 6g, h) and A2780 cells (Supplementary Fig. 6i, j). Altogether, our data proved that FBXO16 accelerated ULK1 degradation by enhancing its K48-linked poly-ubiquitination.

### Clinical *MIR937* amplification correlates with FBXO16 and ULK1 expression

**Fig. 7:**
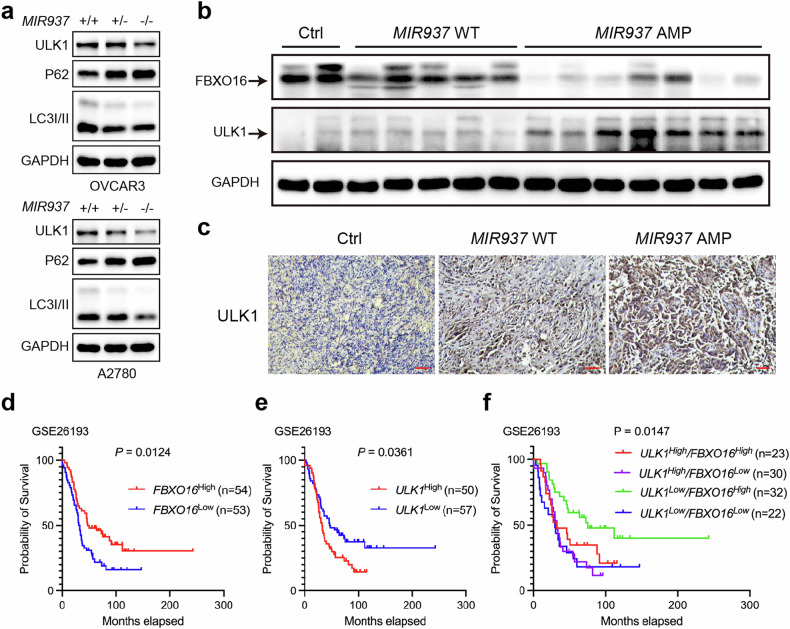
Clinical MIR937 amplification correlates with FBXO16 and ULK1 expression. **a** IB analysis of protein levels for ULK1, P62, LC3II/I in *MIR937*^+/+^, *MIR937*^+/−^, and *MIR937*^−/−^ OVCAR3 (upper) and A2780 (lower) cells. GAPDH was used as loading control. **b** IB analysis for comparing FBXO16, ULK1 protein abundances in clinical OV with control samples. Ctrl: normal ovary, *MIR937* WT: OV patient samples without amplification of *MIR937*. *MIR937* AMP: OV patient samples with *MIR937* amplification. **c** Representative images for IHC analysis of ULK1 protein in OV patient samples or the normal control, Scale bar: 100 μm. Kaplan–Meier survival analysis of OV patients from GSE26193 dataset grouped by FBXO16 (**d**) and ULK1 (**e**) expression. **f** Kaplan–Meier survival analysis of OV patients grouped by both FBXO16 and ULK1 expression in GSE26193. Similar results were obtained in three independent experiments.

We then asked whether *MIR937* amplification in OV functions through FBXO16/ULK1 axis. Firstly, level of ULK1 and autophagy (manifested by p62 and LC3II/I) were detected by western blot in *MIR937* loss OVCAR3 and A2780 cells, and the results showed both ULK1 and autophagy levels were slightly downregulated in *MIR937*^+/−^ cells, while notably decreased in *MIR937*^−/−^ cells in comparison with that of *MIR937*^+/+^ cells (Fig. 7a). To further clarify the dependence of miR-937-5p by *MIR937* loss in inhibiting autophagy, miR-937-5p expression was enhanced and inhibited by mimics and inhibitors, and consistently we found miR-937-5p overexpression promoted cell autophagy, while its knockdown inhibited cell autophagy (Supplementary Fig. 7a). In addition, we checked the ULK1 expression in xenograft tumors derived from *MIR937*^-/-^ OVCAR3 and A2780 cells, which were interfered with agomiR-937-5p or 3p. The results showed agomiR-937-5p restored ULK1 expression by *MIR937* loss (Supplementary Fig. 7b). Furthermore, we also tested whether miR-937-5p effects on ULK1 and autophagy was through its downregulation of FBXO16. So OVCAR3 and A2780 cells were firstly transfected with miR-937-5p inhibitors to transiently increase FBXO16, and then with siRNA targeting FBXO16, to detect whether FBXO16 knockdown could rescue ULK1 and autophagy, which had been suppressed by miR-937-5p inhibition mediated FBXO16 elevation. The results revealed that the restrain on ULK1 and autophagy by miR-937-5p inhibitors could be both ameliorated by FBXO16 knockdown (Supplementary Fig. 7c). So, we concluded that *MIR937* functioned through miR-937-5p/FBXO16/ULK1 to regulate OV cancer progression.

We next examined in clinical whether *MIR937* amplification was correlated with FBXO16 and ULK1 in OV patients. In our study, seven out of all the enrolled HGSOC patients (*n* = 45) were validated with *MIR937* amplification. And the representative data of FBXO16 and ULK1 protein expression in *MIR937* amplified patients was presented in comparison with that of normal or *MIR937* diploid patients. The results indicated that FBXO16 expression was relatively high in normal ovary tissues, slightly reduced in *MIR937* diploid OV samples, while decreased dramatically in *MIR937* amplified patients (Fig. 7b). And inversely, ULK1 expression increased somewhat in *MIR937* diploid OV samples, but was further augmented in *MIR937* amplified patients (Fig. 7b). The results were also validated by IHC assay that ULK1 abundance was reinforced more by *MIR937* amplification than *MIR937* diploid in HGSOC patients (Fig. 7c). Finally, we reviewed the clinical significance for FBXO16 downregulation or ULK1 upregulation in HGSOC patients. For lacking of clinical samples in hand, we take the advantage of Kaplan plotter database to investigate FBXO16 and ULK1 correlation with prognosis of HGSOC patients. Individually, ULK1 high expression predicted worse survival in GSE3149 dataset, while FBXO16 high expression favored HGSOC patient survival in GSE63885 dataset (Supplementary Fig. 7d, e). Moreover, survival analysis by GSE26193 datasets showed the same results (Fig. 7d, e). And when the patients from GSE26193 dataset were grouped according to both FBXO16 and ULK1 expression, we can see that the survival rate for ULK1^low^/FBXO16^high^ patients were greatly improved, while ULK1^high^/FBXO16^low^ patients exhibited the worst survival capabilities (Fig. 7f). Collectively, our study implied that *MIR937* amplification induced ULK1 high expression and FBXO16 low expression simultaneously contribute to malignant progression of HGSOC patients. Finally, to consolidate the clinical relevance and shed light on the precise therapeutic prospect in accordance with *MIR937* CNAs and FBXO16 expression, we divided HGSOC patients into chemotherapy sensitive and insensitive groups from TCGA database. By correlation analysis, we found in the complete/partial response group, *MIR937* CNAs showed no correlation with FBXO16, while in the stable/progressive OV group, *MIR937* CNAs negatively correlated with FBXO16, which implied that the combination of *MIR937* CNAs and FBXO16 expression might be a good indicator of sensitivity to chemotherapy for OV patients (Supplementary Fig. 7f, g).

## Discussion

The data presented here demonstrate that *MIR937* copy number changes functioned through miR-937-5p to dictated autophagy and cell proliferation of HGSOC cells. *MIR937* deficiency abolished miR-937-5p inhibitory effect on FBXO16, resulted in accumulation of ULK1 protein, and enhanced autophagy, thereby facilitating OV cell proliferation and accelerating tumor progression. Mechanistically, *MIR937* copy number amplification upregulated miR-937-5p, which strengthened its binding with 3′ UTR of FBXO16, and attenuating FBXO16 mediated ULK1 degradation via SCF^FBXO16^ complex. Importantly, FBXO16 is downregulated and ULK1 is upregulated in *MIR937* amplified HGSOC samples, and FBXO16^high^ULK1^low^ favors survival for HGSOC patients. Collectively, these results unveiled a regulatory axis for promoting OV carcinogenesis elicited by *MIR937* amplification.

Somatic CNAs are hallmarks of many cancers [27–29]. Somatic CNAs generally refers to gain or loss of large fragments of genomic DNA. From this sight, CNAs of a single genes did not always occur exclusively with that of its adjacent genes. From this point, the oncogenic or the tumor suppressive effects of amplification or lost CNAs might be a combinational result for a cluster of genes. The matter is the same for miRNAs despite their minimal nucleotides in length. Although tumorigenesis function of CNAs for protein-coding genes has been extensively studied, the studies for miRNAs CNAs were still limited which hindered the processes for precise targeting of patients with specific alteration. Using TCGA database for systematic analysis, we observed that *MIR937* amplification correlated with enhanced proliferation with HGSOC tumors. Correspondingly, deletion of *MIR937* in HGSOC cells greatly restricted cell proliferation and tumor formation in mouse xenograft models. Importantly, homo-loss of *MIR937* exacerbated the inhibitory effects exerted by heterozygous loss of *MIR937* for tumor growth. These results identify a dose effect of *MIR937* copy number in tumor promotion.

By rescue and bioinformatics analysis, we confirmed miR-937-5p is responsible for *MIR937* oncogenic roles and interfered with FBXO16 function. FBXO16 belongs to F-box protein that participates in the assembly of cullin-RING ubiquitin ligase (CRL) [30]. A typical member of CRL is SKP1/CUL1/F-box (SCF) E3 ubiquitin ligase complex, which controlled protein turnover in a proteasome-dependent manner. Generally, SKP1 and F-box protein act as adapter, and recruit the substrate to CUL1, where RBX1 promotes poly-ubiquitination of the targeted protein [31]. Due to alternative cullin members and diversity of F-box proteins, nearly 200 unique CRLs existed in the eukaryotic cells [32]. Although FBXO16 has been revealed to suppress OV progression [33]; how *MIR937* amplification directs FBXO16 function in HGSOC and whether *MIR937* attenuated FBXO16 containing SCF activity remains elusive. Our experiments showed that *MIR937* deficiency highly increased FBXO16 expression, and upon mutation of its putative binding site in 3′ UTR of FBXO16, the inhibition on FBXO16 expression was diminished. Notably, 4D label-free quantitative proteomics study revealed that FBXO16 might negatively regulate autophagy related pathways, and knockdown of FBXO16 restored autophagy in OV cells suppressed by miR-937-5p inhibitors. Based on these findings, we hypothesized *MIR937* amplification improve autophagy though FBXO16. Indeed, we found FBXO16 interacted with ULK1 and accelerated its degradation by promote its K48 linked poly-ubiquitination. Further, our data also defined the interaction between ULK1 and SKP1, CUL1, and RBX1. So, we propose that *MIR937* amplification function through SCF^FBXO16^ to modulate ULK1 mediated autophagy.

Autophagy is a complicated process that is capable of recycling intracellular components to maintain metabolism and survival. Recent studies assumed that autophagy might play opposite roles in cancer to suppress initiation, while promote growth and progression for established tumors [34, 35]. So it is delicate in targeting autophagy for cancer treatment. In HGSOC, autophagy has been reported to promote metastasis and chemo-resistance [36], and inhibition of autophagy might induce apoptosis [37]. Besides, ULK1 inhibitor can reduce autophagy and then viability of HGSOC cells [38]. In our study, we found *MIR937* positively regulated ULK1-mediated autophagy and ULK1 protein accumulation is accompanied with reduced FBXO16 protein and *MIR937* amplification in HGSOC patient samples. In particular, clinical data revealed patients expressing with high FBXO16 and low ULK1 favors better prognosis. Collectively, our data provided an alternative therapeutic strategy targeting ULK1-autophagy in *MIR937* amplified HGSOC patients.

In summary, our findings identify MIR937 amplification as an oncogenic feature of HGSOC patients. It not only defines the fundamental effects of *MIR937* in tumor promotion in mouse models, but also highlights the correlation of *MIR937* amplification and ULK1 mediated autophagy in HGSOC. The results suggest that targeting of miR-937-5p holds great prosperities for treatment of HGSOC with *MIR937* amplification.

## Supplementary information

Supplementary material

Uncropped WB

## Data Availability

The supporting data for the findings of this study can be obtained from the corresponding author upon reasonable request.

## References

[1] Torre LA, Trabert B, DeSantis CE, Miller KD, Samimi G, Runowicz CD, et al. Ovarian cancer statistics, 2018. CA Cancer J Clin. 2018;68:284–96. 29809280 10.3322/caac.21456 PMC6621554

[2] Vaughan S, Coward JI, Bast RC Jr., Berchuck A, Berek JS, Brenton JD, et al. Rethinking ovarian cancer: recommendations for improving outcomes. Nat Rev Cancer. 2011;11:719–25. 21941283 10.1038/nrc3144 PMC3380637

[3] Cannistra SA. Cancer of the ovary. N Engl J Med. 2004;351:2519–29. 15590954 10.1056/NEJMra041842

[4] Matsuo K, Machida H, Matsuzaki S, Grubbs BH, Klar M, Roman LD, et al. Evolving population-based statistics for rare epithelial ovarian cancers. Gynecol Oncol. 2020;157:3–11. 31954534 10.1016/j.ygyno.2019.11.122 PMC7526050

[5] Jacobs IJ, Menon U, Ryan A, Gentry-Maharaj A, Burnell M, Kalsi JK, et al. Ovarian cancer screening and mortality in the UK collaborative trial of ovarian cancer screening (UKCTOCS): a randomised controlled trial. Lancet. 2016;387:945–56. 26707054 10.1016/S0140-6736(15)01224-6 PMC4779792

[6] Beroukhim R, Mermel CH, Porter D, Wei G, Raychaudhuri S, Donovan J, et al. The landscape of somatic copy-number alteration across human cancers. Nature. 2010;463:899–905. 20164920 10.1038/nature08822 PMC2826709

[7] Henrichsen CN, Vinckenbosch N, Zöllner S, Chaignat E, Pradervand S, Schütz F, et al. Segmental copy number variation shapes tissue transcriptomes. Nat Genet. 2009;41:424–9. 19270705 10.1038/ng.345

[8] Stranger BE, Forrest MS, Dunning M, Ingle CE, Beazley C, Thorne N, et al. Relative impact of nucleotide and copy number variation on gene expression phenotypes. Science. 2007;315:848–53. 17289997 10.1126/science.1136678 PMC2665772

[9] Stingele S, Stoehr G, Peplowska K, Cox J, Mann M, Storchova Z. Global analysis of genome, transcriptome, and proteome reveals the response to aneuploidy in human cells. Mol Syst Biol. 2012;8:608. 22968442 10.1038/msb.2012.40 PMC3472693

[10] Tang YC, Amon A. Gene copy-number alterations: a cost-benefit analysis. Cell. 2013;152:394–405. 23374337 10.1016/j.cell.2012.11.043 PMC3641674

[11] Theurillat JP, Metzler SC, Henzi N, Djouder N, Helbling M, Zimmermann AK, et al. URI is an oncogene amplified in ovarian cancer cells and is required for their survival. Cancer Cell. 2011;19:317–32. 21397856 10.1016/j.ccr.2011.01.019

[12] Han C, Yang L, Choi HH, Baddour J, Achreja A, Liu Y, et al. Amplification of USP13 drives ovarian cancer metabolism. Nat Commun. 2016;7:13525. 27892457 10.1038/ncomms13525 PMC5133706

[13] Deng P, Wang Z, Chen J, Liu S, Yao X, Liu S, et al. RAD21 amplification epigenetically suppresses interferon signaling to promote immune evasion in ovarian cancer. J Clin Invest. 2022;132:e159628. 10.1172/JCI159628 PMC9663158 36201246

[14] Baselga J, Norton L, Albanell J, Kim YM, Mendelsohn J. Recombinant humanized anti-HER2 antibody (Herceptin) enhances the antitumor activity of paclitaxel and doxorubicin against HER2/neu overexpressing human breast cancer xenografts. Cancer Res. 1998;58:2825–31. 9661897

[15] Esquela-Kerscher A, Slack FJ. Oncomirs—microRNAs with a role in cancer. Nat Rev Cancer. 2006;6:259–69. 16557279 10.1038/nrc1840

[16] Bartel DP. MicroRNAs: genomics, biogenesis, mechanism, and function. Cell. 2004;116:281–97. 14744438 10.1016/s0092-8674(04)00045-5

[17] Medina PP, Nolde M, Slack FJ. OncomiR addiction in an in vivo model of microRNA-21-induced pre-B-cell lymphoma. Nature. 2010;467:86–90. 20693987 10.1038/nature09284

[18] Okada N, Lin CP, Ribeiro MC, Biton A, Lai G, He X, et al. A positive feedback between p53 and miR-34 miRNAs mediates tumor suppression. Genes Dev. 2014;28:438–50. 24532687 10.1101/gad.233585.113 PMC3950342

[19] Chaluvally-Raghavan P, Zhang F, Pradeep S, Hamilton MP, Zhao X, Rupaimoole R, et al. Copy number gain of hsa-miR-569 at 3q26.2 leads to loss of TP53INP1 and aggressiveness of epithelial cancers. Cancer Cell. 2014;26:863–79. 25490449 10.1016/j.ccell.2014.10.010 PMC4261159

[20] van Zandwijk N, Pavlakis N, Kao SC, Linton A, Boyer MJ, Clarke S, et al. Safety and activity of microRNA-loaded minicells in patients with recurrent malignant pleural mesothelioma: a first-in-man, phase 1, open-label, dose-escalation study. Lancet Oncol. 2017;18:1386–96. 28870611 10.1016/S1470-2045(17)30621-6

[21] Smith ES, Whitty E, Yoo B, Moore A, Sempere LF, Medarova Z. Clinical applications of short non-coding RNA-based therapies in the era of precision medicine. Cancers*.* 2022;14:1588. 10.3390/cancers14061588 PMC8946086 35326738

[22] Soh J, Cho H, Choi CH, Lee H. Identification and characterization of microRNAs associated with somatic copy number alterations in cancer. Cancers*.* 2018;10:475. 10.3390/cancers10120475 PMC6315597 30501131

[23] Marcinkowska M, Szymanski M, Krzyzosiak WJ, Kozlowski P. Copy number variation of microRNA genes in the human genome. BMC Genom. 2011;12:183. 10.1186/1471-2164-12-183 PMC3087710 21486463

[24] Zhang Z, Zhang L, Wang B, Wei R, Wang Y, Wan J, et al. MiR-337-3p suppresses proliferation of epithelial ovarian cancer by targeting PIK3CA and PIK3CB. Cancer Lett. 2020;469:54–67. 31629932 10.1016/j.canlet.2019.10.021

[25] Goode EL, Chenevix-Trench G, Song H, Ramus SJ, Notaridou M, Lawrenson K, et al. A genome-wide association study identifies susceptibility loci for ovarian cancer at 2q31 and 8q24. Nat Genet. 2010;42:874–9. 20852632 10.1038/ng.668 PMC3020231

[26] Bell D, Berchuck A, Birrer M, Chien J, Cramer DW, Dao F, et al. Integrated genomic analyses of ovarian carcinoma. Nature. 2011;474:609–15. 21720365 10.1038/nature10166 PMC3163504

[27] Steele CD, Abbasi A, Islam SMA, Bowes AL, Khandekar A, Haase K, et al. Signatures of copy number alterations in human cancer. Nature. 2022;606:984–91. 35705804 10.1038/s41586-022-04738-6 PMC9242861

[28] Zack TI, Schumacher SE, Carter SL, Cherniack AD, Saksena G, Tabak B, et al. Pan-cancer patterns of somatic copy number alteration. Nat Genet. 2013;45:1134–40. 24071852 10.1038/ng.2760 PMC3966983

[29] Jakubek YA, Chang K, Sivakumar S, Yu Y, Giordano MR, Fowler J, et al. Large-scale analysis of acquired chromosomal alterations in non-tumor samples from patients with cancer. Nat Biotechnol. 2020;38:90–96. 31685958 10.1038/s41587-019-0297-6 PMC8082517

[30] Paul D, Islam S, Manne RK, Dinesh US, Malonia SK, Maity B, et al. F-box protein FBXO16 functions as a tumor suppressor by attenuating nuclear β-catenin function. J Pathol. 2019;248:266–79. 30714168 10.1002/path.5252 PMC6619347

[31] Bennett EJ, Rush J, Gygi SP, Harper JW. Dynamics of cullin-RING ubiquitin ligase network revealed by systematic quantitative proteomics. Cell. 2010;143:951–65. 21145461 10.1016/j.cell.2010.11.017 PMC3008586

[32] Petroski MD, Deshaies RJ. Function and regulation of cullin-RING ubiquitin ligases. Nat Rev Mol Cell Biol. 2005;6:9–20. 15688063 10.1038/nrm1547

[33] Ji M, Zhao Z, Li Y, Xu P, Shi J, Li Z, et al. FBXO16-mediated hnRNPL ubiquitination and degradation plays a tumor suppressor role in ovarian cancer. Cell Death Dis. 2021;12:758. 34333526 10.1038/s41419-021-04040-9 PMC8325689

[34] Amaravadi R, Kimmelman AC, White E. Recent insights into the function of autophagy in cancer. Genes Dev. 2016;30:1913–30. 27664235 10.1101/gad.287524.116 PMC5066235

[35] White E. Deconvoluting the context-dependent role for autophagy in cancer. Nat Rev Cancer. 2012;12:401–10. 22534666 10.1038/nrc3262 PMC3664381

[36] Ma H, Li Y, Wang X, Wu H, Qi G, Li R, et al. PBK, targeted by EVI1, promotes metastasis and confers cisplatin resistance through inducing autophagy in high-grade serous ovarian carcinoma. Cell Death Dis. 2019;10:166. 30778048 10.1038/s41419-019-1415-6 PMC6379381

[37] Salvi A, Young AN, Huntsman AC, Pergande MR, Korkmaz MA, Rathnayake RA, et al. PHY34 inhibits autophagy through V-ATPase V0A2 subunit inhibition and CAS/CSE1L nuclear cargo trafficking in high grade serous ovarian cancer. Cell Death Dis. 2022;13:45. 35013112 10.1038/s41419-021-04495-w PMC8748433

[38] Singha B, Laski J, Ramos Valdés Y, Liu E, DiMattia GE, Shepherd TG. Inhibiting ULK1 kinase decreases autophagy and cell viability in high-grade serous ovarian cancer spheroids. Am J Cancer Res. 2020;10:1384–99. 32509386 PMC7269771

