# Supplementary material for: MIR937 amplification potentiates ovarian cancer progression by attenuating FBXO16 inhibition on ULK1-mediated autophagy
Source: Cell Death Dis. 2024 Oct 9;15(10):735. doi: 10.1038/s41419-024-07120-8 (PMC11464496; doi:10.1038/s41419-024-07120-8)
Supplement: Supplementary file 1 — Supplementary material [file 41419_2024_7120_MOESM1_ESM.docx]

####
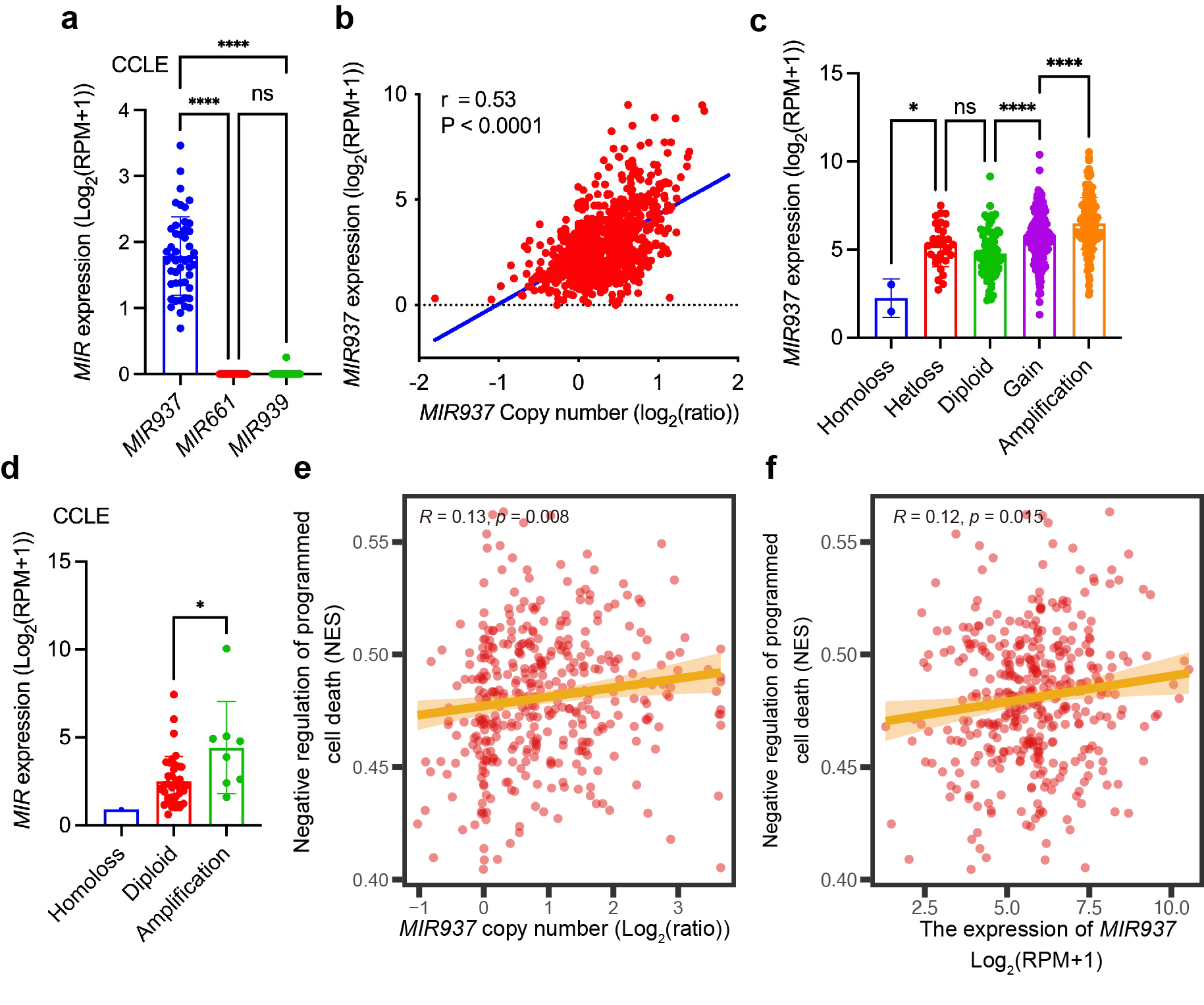


#### Supplementary Fig. 1. *MIR937* copy number correlated with its expression and death related pathway of HGSOC. a, The expression data of three miRs were retrieved from CCLE OV cancer cell lines. b, The correlations between *MIR937* copy number and its expression were performed by the data from CCEL cancer cell lines. c, d, The comparison of *MIR937* expression in HGSOC patients (c) or OV cancer cell lines (d) with the indicated status of *MIR937* genomic alterations. e, Pearson correlation between the *MIR937* copy number and the cell death score calculated by ssGSEA. f, Pearson correlation between the *MIR937* mRNA expression and the cell death score calculated by ssGSEA. The gene set named “negative regulation of programmed cell death” was obtained from the Gene Ontology (GO) knowledgebase (https://www.geneontology.org/). ssGSEA: single sample gene set enrichment analysis. Statistical analysis was performed using one-way ANOVA in a, c and d; Pearson correlation coefficients (R) were employed to evaluate the correlations between two continuous variables in b, e and f. Statistical analysis was performed using one-way ANOVA in a, c, and d; Pearson correlation coefficients (R) were employed to evaluate the correlations between two continuous variables in b, e, and f. **P* < 0.05; *****P* < 0.0001.

####
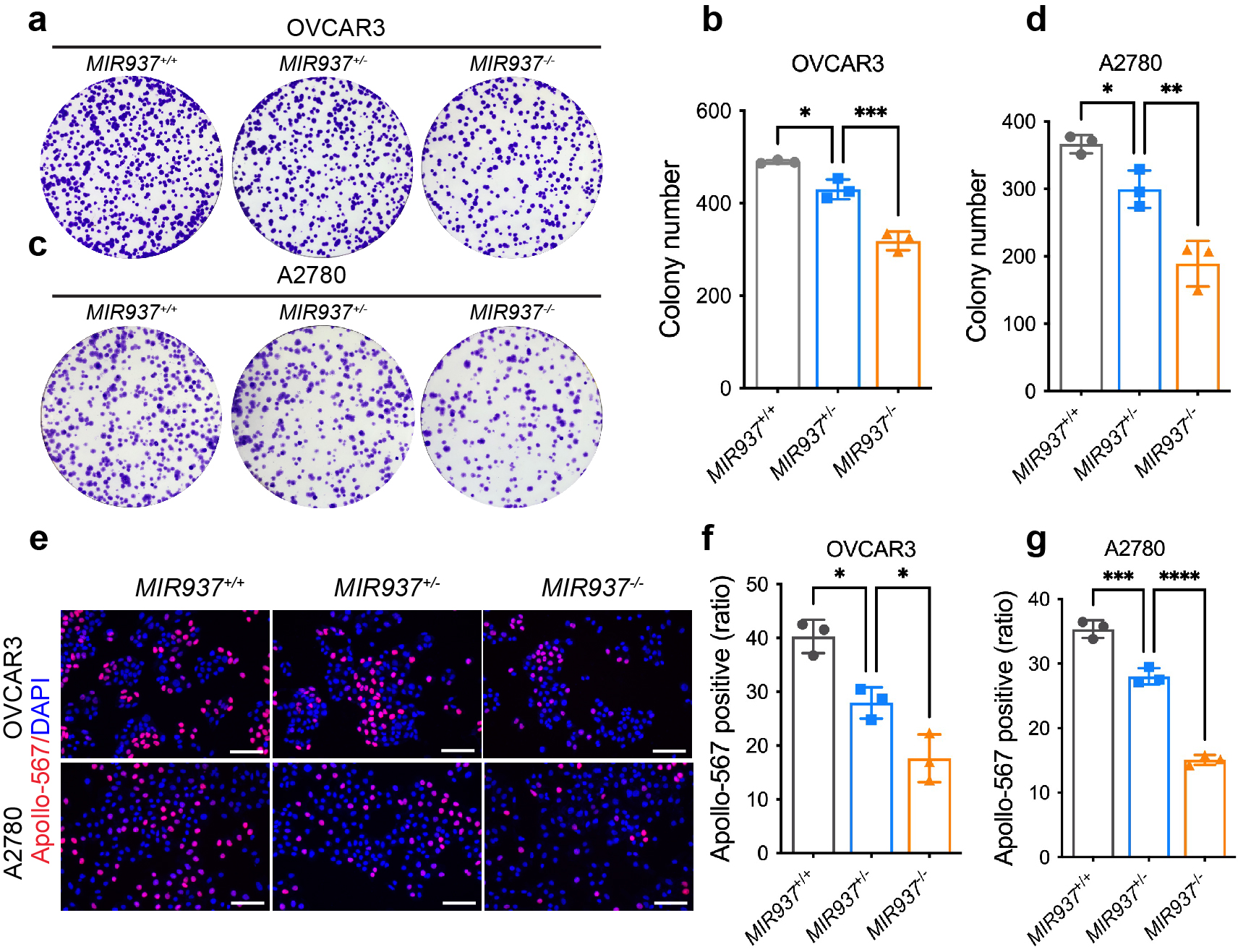


#### Supplementary Fig. 2. *MIR937* gene loss attenuated proliferation of OV cancer cells. a, c, Colony formed by OVCAR3 (a) and A2780 (c) cells stained with crystal violet solution. b, d, The number of colonies for each group of OVCAR3 (b) and A2780 (d) cells were counted and summarized by ImageJ software. e, Different groups of OVCAR3 and A2780 cells were incubated with EdU, and images were captured under microscope. Scale bar: 100 μm. f, g Appolo-567 positive cells were counted and summarized by ImageJ software for OVCAR3 (f) and A2780 (g) cells. Data are shown as the mean ± SD. Similar results were obtained in three independent experiments.Statistical analysis was performed using one-way ANOVA in b, d, f and g; **P* < 0.05. ***P* < 0.01. ****P* < 0.001. *****P* < 0.0001.

####
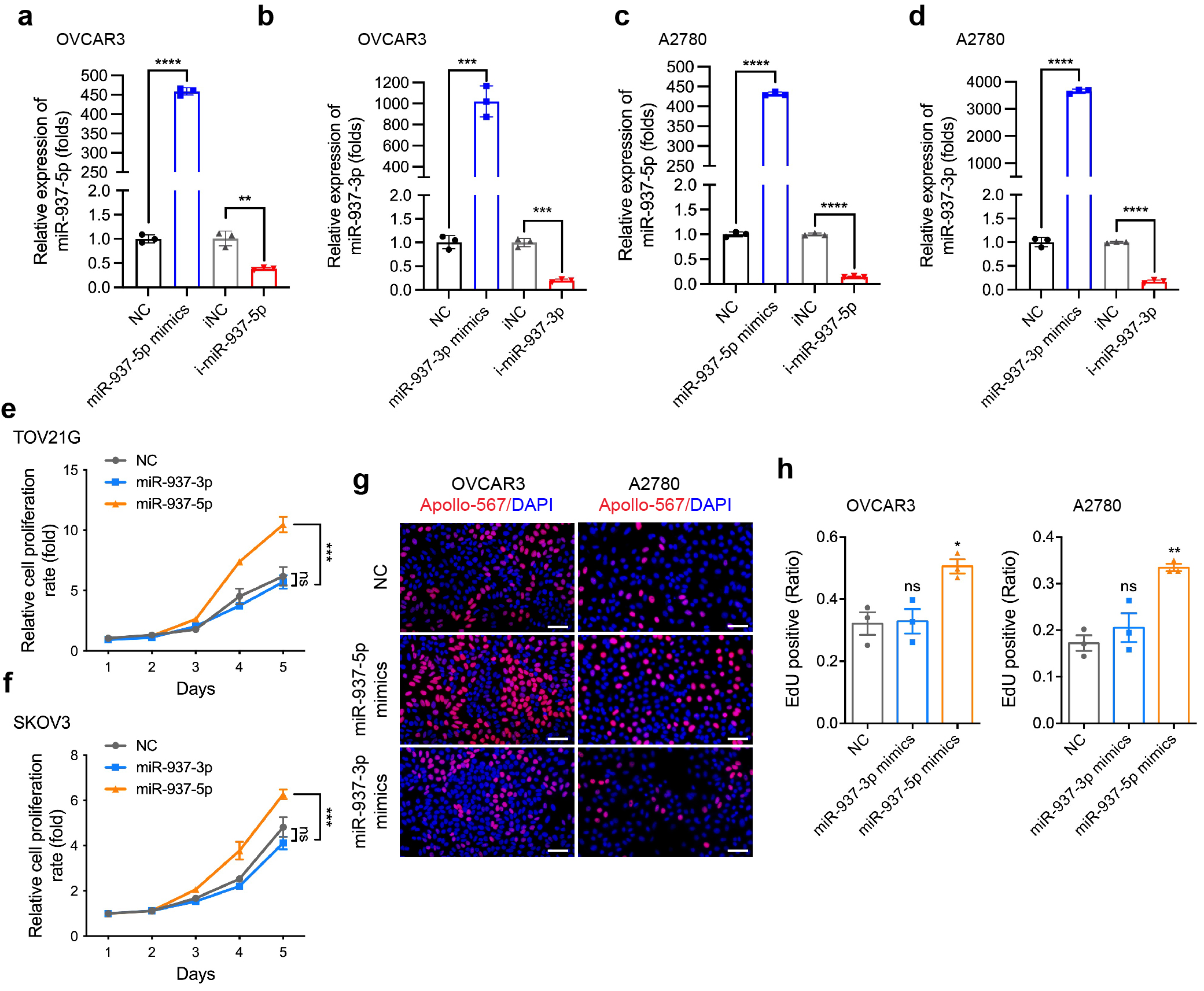


#### Supplementary Fig. 3. miR-937-5p promoted proliferation of OV cancer cells. a-d, The over-expression and knockdown efficiency of miR-937-5p and miR-937-3p was detected by qRT-PCR in OVCAR3 (a and b) and A2780 cells (c and d). The cells were transfected in 12-well dishes with mimics or inhibitors of miR-937-5p or 3p, and the cells were harvested 48 hours post-transfection. e, f, Growth curve for TOV21G (e) and SKOV3 (f) cells were analyzed by CCK8 upon miR-937-5p or 3p mimics transfection. g, EdU assay was performed to visualize the cell ratio for proliferative cells. Scale bar: 100 μm. h, Appolo-567 positive cells were counted and summarized by ImageJ software for OVCAR3 and A2780 cells. And the images were captured 48 hours’ post-transfection of miR-937-5p and 3p mimics. Data are shown as the mean ± SD. Similar results were obtained in three independent experiments. Statistical analysis was performed using two- tailed Student’s t test in a-d and one-way ANOVA in e, f and h; **P* < 0.05. ****P* < 0.001. *****P* < 0.0001.

####
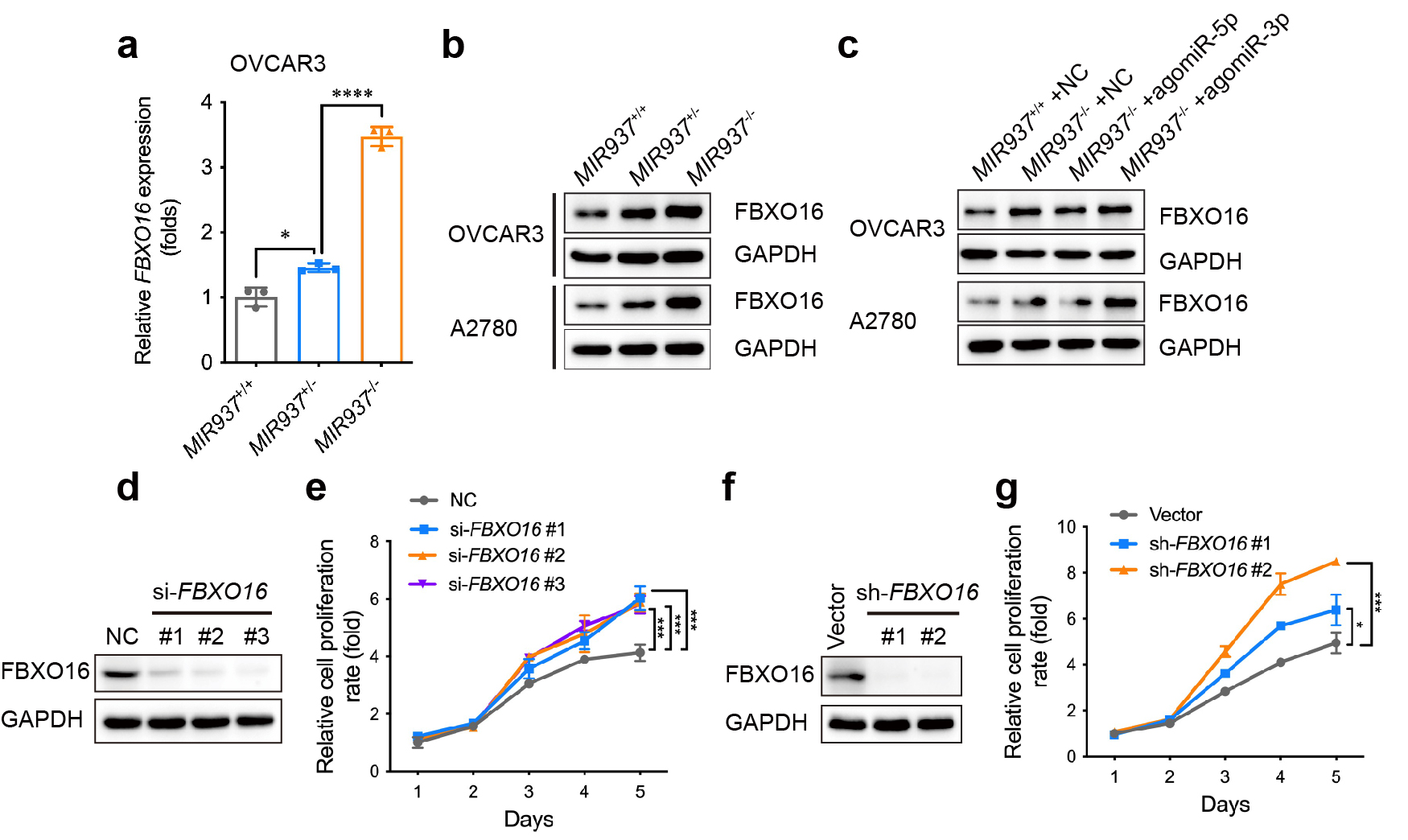


#### Supplementary Fig. 4. miR-937-5p deficiency by *MIR937* loss enhanced FBXO16 expression. a, qRT-PCR detection of FBXO16 expression in OVCAR3 cells with different grade of *MIR937* gene loss. b, IB analysis of FBXO16 expression in OVCAR3 and A2780 cells with or without *MIR937* loss. c, IB analysis of FBXO16 expression in xenograft tumors derived from OVCAR3 and A2780 (*MIR937*^+/+^, *MIR937*^-/-^) cells, which were interfered with agomiR-937-5p or 3p injection. d, IB analysis of FBXO16 knockdown efficiency in OVCAR3 by siRNAs. e, Growth curve analysis for OVCARA3 cells transfected with three siRNAs targeting FBXO16. f, IB analysis of silenced FBXO16 protein expression in shRNA stably expressed OVCAR3 cells. g, Growth curve analysis for OVCAR3 cells with stable knockdown of FBXO16. Data are shown as the mean ± SD. Similar results were obtained in three independent experiments. Statistical analysis was performed using one-way ANOVA in a, e and g; **P* < 0.05. ****P* < 0.001.

####
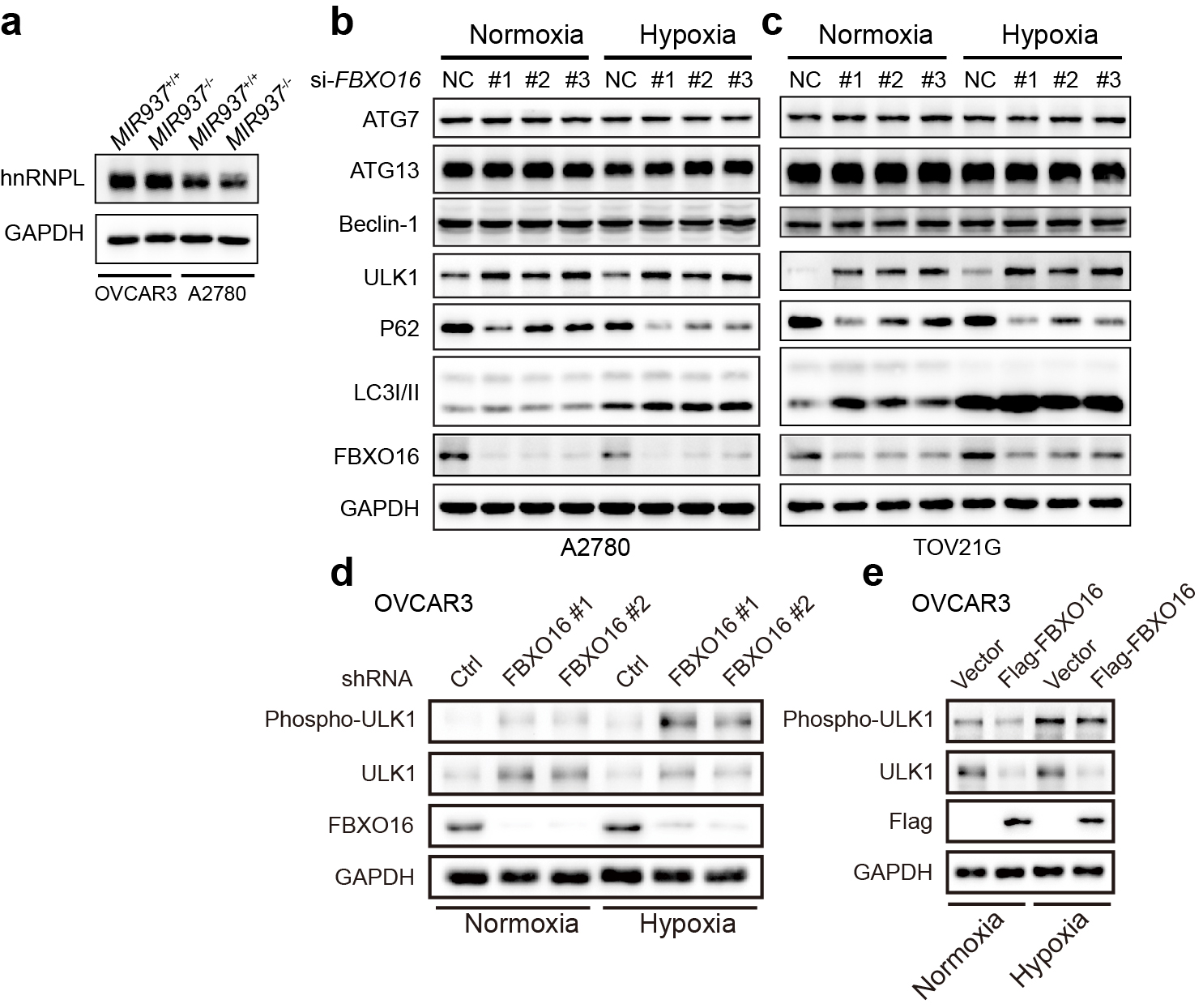


#### Supplementary Fig. 5. FBXO16 knockdown increased ULK1 expression and autophagy. a, IB analysis of protein level for hnRNPL in *MIR937*^+/+^ and *MIR937*^-/-^ OVCAR3 and A2780 cells. b, c, IB analysis for autophagy related proteins affected by transient knockdown of FBXO16 in A2780 (**b**) and TOV21G (c) cells under normal and hypoxia conditions. NC and siRNA were transfected with lipofectamine 2000, and 48 hours’ post-transfection, the cells were treated under hypoxia condition with 1% oxygen at 37˚C for 12 hours. **d**, e, IB analysis for phospho-ULK1 (Thr180) affected by stable knock-down (**d**) and over-expression (**e**) of FBXO16 in OVCAR3 cells under normal and hypoxia conditions. Similar results were obtained in three independent experiments.


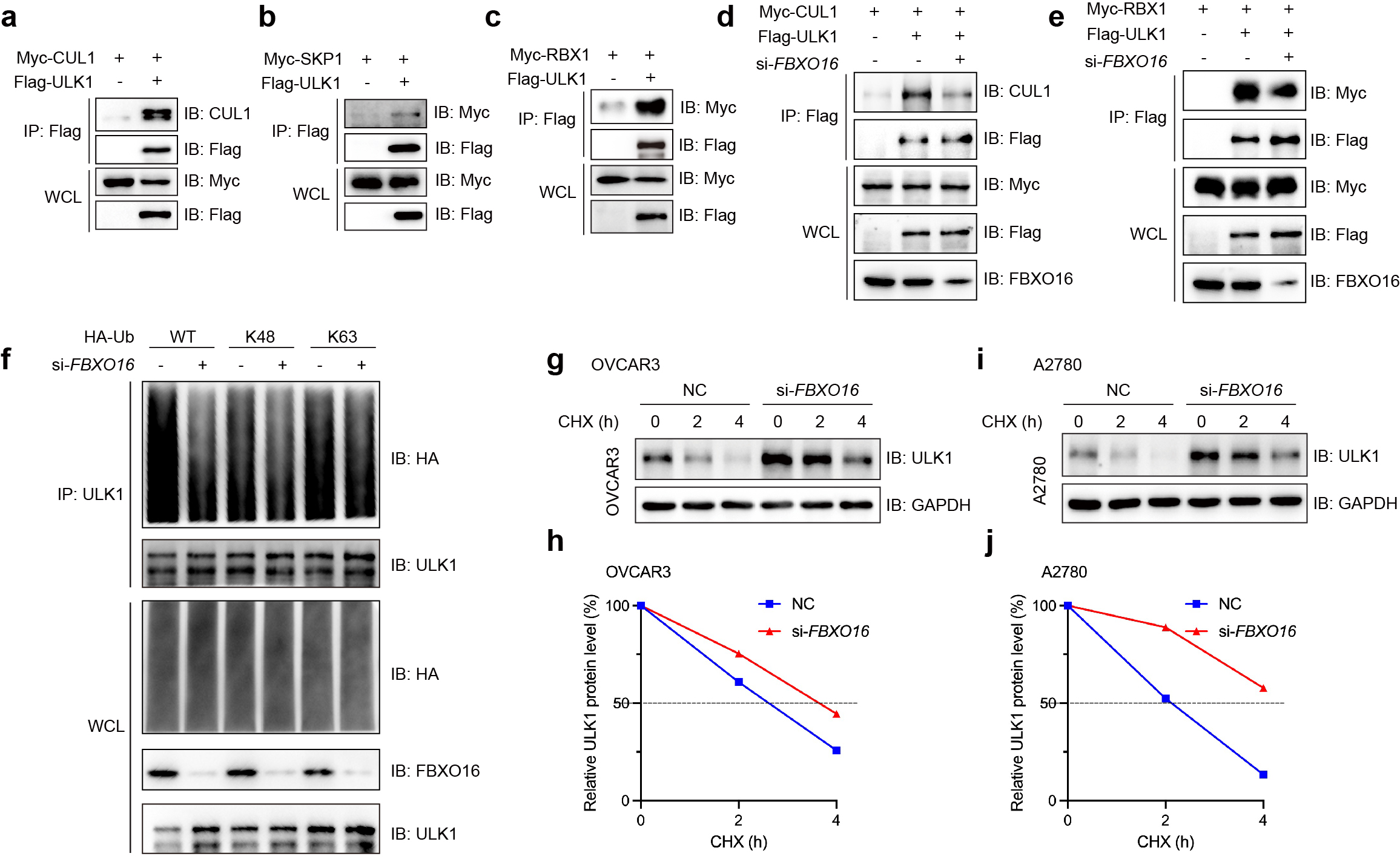


#### Supplementary Fig. 6. ULK1 interacted with CUL1-SKP1-RBX1 complex, and thus inhibited its stability. a-c, HEK293T cell were transfected with the indicated plasmids. Cell lysates were subjected to Co-IP analysis with anti-Flag antibody to detect Flag-ULK1 interaction with Myc-CUL1 (a), Myc-SKP1 (b), and Myc-RBX1 (c). **d,e,** Co-IP analysis with anti-Flag antibody to detect the interaction between Flag-ULK1 and Myc-CUL1 (**d**), and Myc-RBX1 (**e**) in HEK293T cells co-transfected with siRNA targeting FBXO16. **f,** Co-IP with anti-ULK1 antibody followed by IB analysis to detect ULK1 ubiquitination affected by FBXO16 knockdown in OVCAR3 cells. WT, K48, and K63 mutant forms of HA-Ub encoding plasmids were transfected into OVCAR3 cells, respectively. g-j, IB analysis of ULK1 protein in OVCAR3 (**g**) and A2780 (**i**) cells treated with cycloheximide (CHX) for the indicated times upon FBXO16 knockdown, and ULK1 protein levels were quantified by measuring band intensities and normalized to GAPDH for OVCAR3 (**h**) and A2780 (**j**) cells. Similar results were obtained in three independent experiments.

####
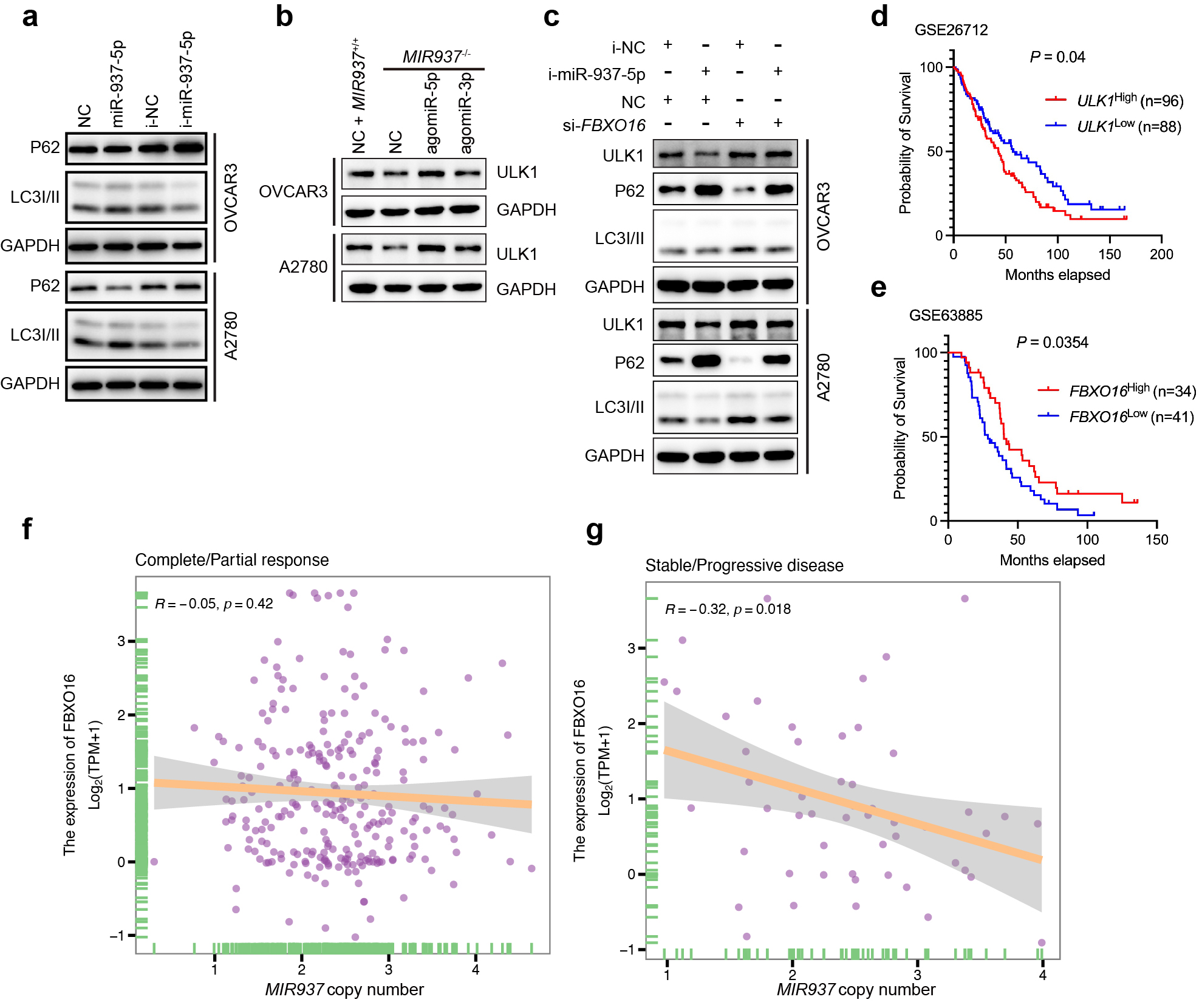


#### Supplementary Fig. 7. miR-937-5p enhanced autophagy through FBXO16/ULK1. a, IB analysis for P62 and LC3II/I in OVCAR3 and A2780 cells transfected with mimics or inhibitors of miR-937-5p and miR-937-3p. b, ULK1 protein was detected in xenograft tumors derived from *MIR937*^+/+^, and *MIR937*^-/-^ cells (OVCAR3, and A2780), which were interfered with agomiR-937-5p and agomiR-937-3p. c, The cells were firstly transfected with miR-937-5p inhibitors, and then transfected with siRNA targeting FBXO16 in OVCAR3 and A2780 cells. IB assay was performed for ULK1, P62, LC3II/I. d, e, Kaplan-Meier survival analysis of OV patients from GSE26712 (d) and GSE63885 (e) were performed individually for ULK1 and FBOX16. Similar results were obtained in three independent experiments. **f,** Pearson’s correlation between the CNA of *MIR937* and RNA expression for gene FBXO16 among the TCGA HGSOC patients exhibiting complete or partial response. g, Pearson’s correlation between the CNA of MIR937 and RNA expression for gene FBXO16 among the TCGA HGSOC patients exhibiting stable or progressive disease.

Supplementary Table 1. Oligo sequences used in the experiments.

| **Oligo ID** | **Sequence (5’-3’)** |
| --- | --- |
| Mimic negative control | Sense: UUCUCCGAACGUGUCACGUTT  Antisense: ACGUGACACGUUCGGAGAATT |
| MiR-937-5p mimic | Sense: GUGAGUCAGGGUGGGGCUGG  Antisense: CCAGCCCCACCCUGACUCAC |
| MiR-937-3p mimic | Sense: AUCCGCGCUCUGACUCUCUGCC  Antisense: GGCAGAGAGUCAGAGCGCGGAU |
| Inhibitor negative control | CAGUACUUUUGUGUAGUACAA |
| MiR-937-5p inhibitor | CCAGCCCCACCCUGACUCAC |
| MiR-937-3p inhibitor | GGCAGAGAGUCAGAGCGCGGAU |
| FBXO16-siRNA#1 | Sense: GGCAAAUGGUUUGACAAAUTT  Antisense: AUUUGUCAAACCAUUUGCCTT |
| FBXO16-siRNA#2 | Sense: CAAGGGUGUUAUCUUUAUATT  Antisense: UAUAAAGAUAACACCCUUGTT |
| FBXO16-siRNA#3 | Sense: GACAGUCACAUGAUAAGAATT  Antisense: UUCUUAUCAUGUGACUGUCTT |

Supplementary Table 2. Oligo sequences used for qRT-PCR analysis.

| **Gene symbol** | **Sequence (5’-3’)** |
| --- | --- |
| BRD7 | Sense: AAGCACAAGTCGGACAAACAC  Antisense: CGTTCCCTCCTACTTTGAGGAC |
| CHEK1 | Sense: ATATGAAGCGTGCCGTAGACT  Antisense: TGCCTATGTCTGGCTCTATTCTG |
| FBXO16 | Sense: AGGATGGATTTGTAATCGCTGAC  Antisense: CGAAAAGCTGATAAAGGGGACT |
| FBXO32 | Sense: GCCTTTGTGCCTACAACTGAA  Antisense: CTGCCCTTTGTCTGACAGAAT |
| HTRA1 | Sense: TCCCAACAGTTTGCGCCATAA  Antisense: CCGGCACCTCTCGTTTAGAAA |
| PBOV1 | Sense: AAGGAACCAGAAATATGAGG  Antisense: TTTGGATAAGTAGAGAAGAC |
| PPP2R1B | Sense: CTTGTGTCAGTATTGCCCAGT  Antisense: TGCTGCTTGTCGAAGTGTAGG |
| RASSF8 | Sense: AGTTCAGAGGATTGTTTGTGGAG  Antisense: CACATCACTAGCATACTGCCC |
| SOX7 | Sense: AGCCGGAGCAGACCTTCTT  Antisense: GCCGGGGAGTAATAGGCAG |
| TP53 | Sense: CAGCACATGACGGAGGTTGT  Antisense: TCATCCAAATACTCCACACGC |
| TUSC3 | Sense: GAGTTCCAGACGCTCAATCTTC  Antisense: GCCAGGAGTTCGCCAGTATT |
