# Supplementary material for: MIR937 amplification potentiates ovarian cancer progression by attenuating FBXO16 inhibition on ULK1-mediated autophagy
Source: Cell Death Dis. 2024 Oct 9;15(10):735. doi: 10.1038/s41419-024-07120-8 (PMC11464496; doi:10.1038/s41419-024-07120-8)

Fig. 4e

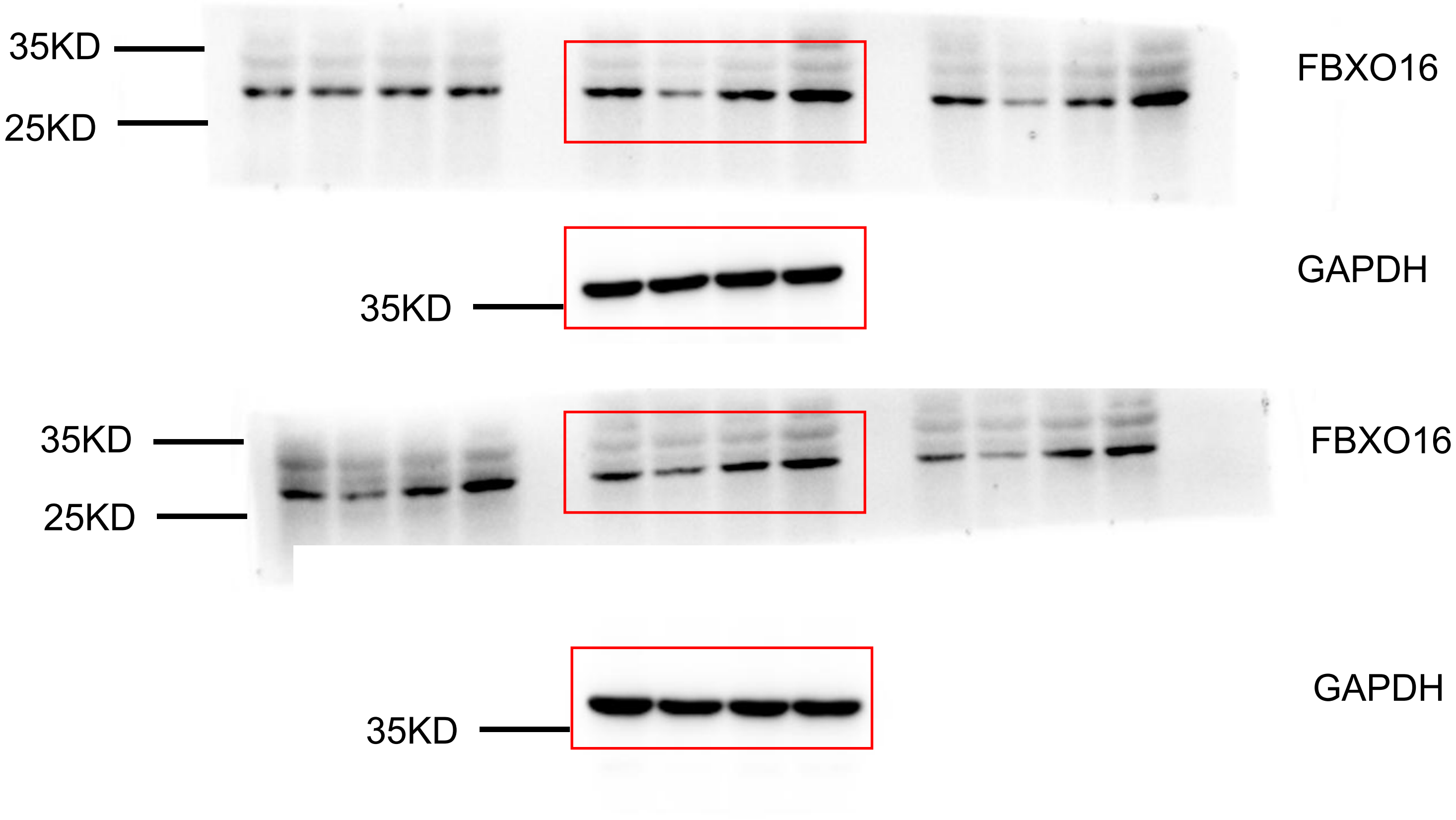

Fig. 5b

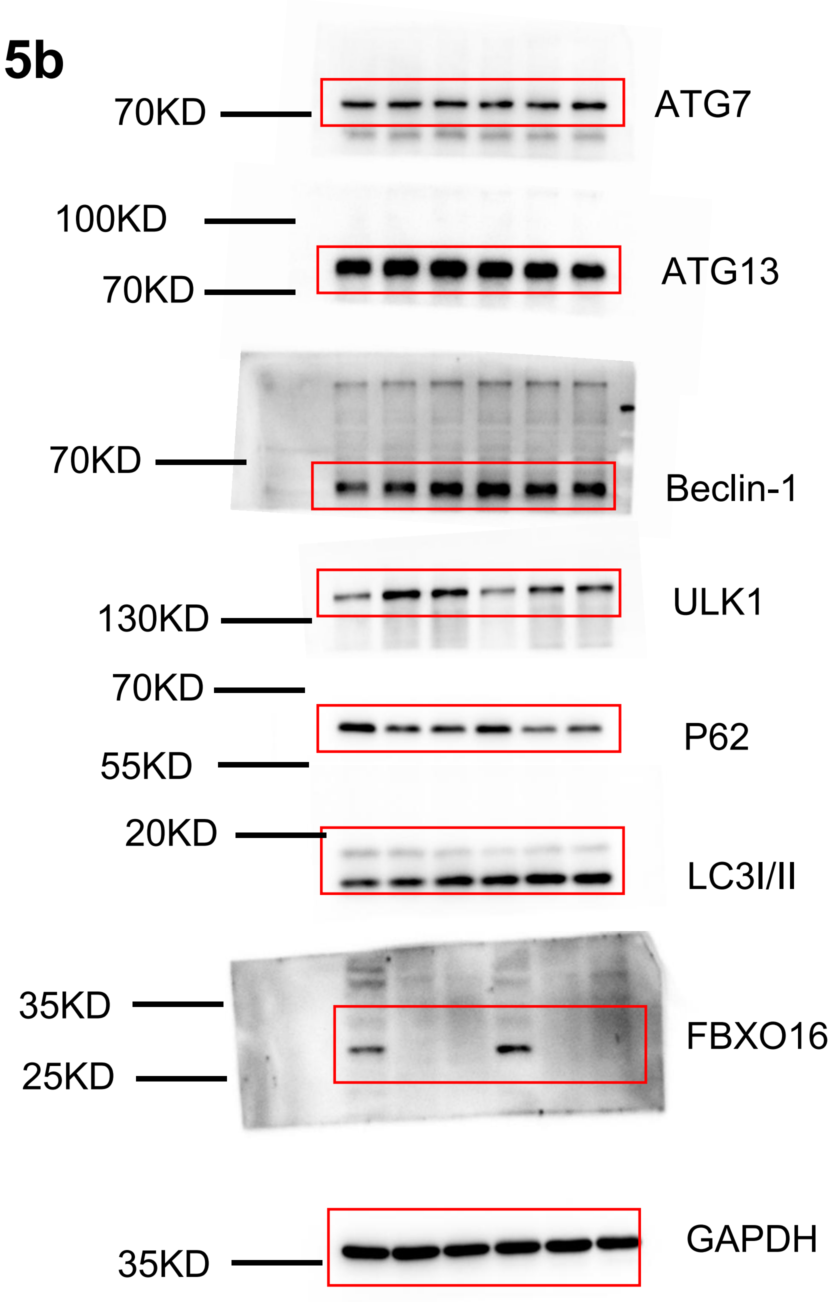
70KD
ATG7
100KD
70KD
ATG13
70KD
Beclin-1
ULK1
130KD
70KD
P62
55KD
20KD
LC3I/II
35KD
FBXO16
25KD
GAPDH
35KD

Fig. 5f

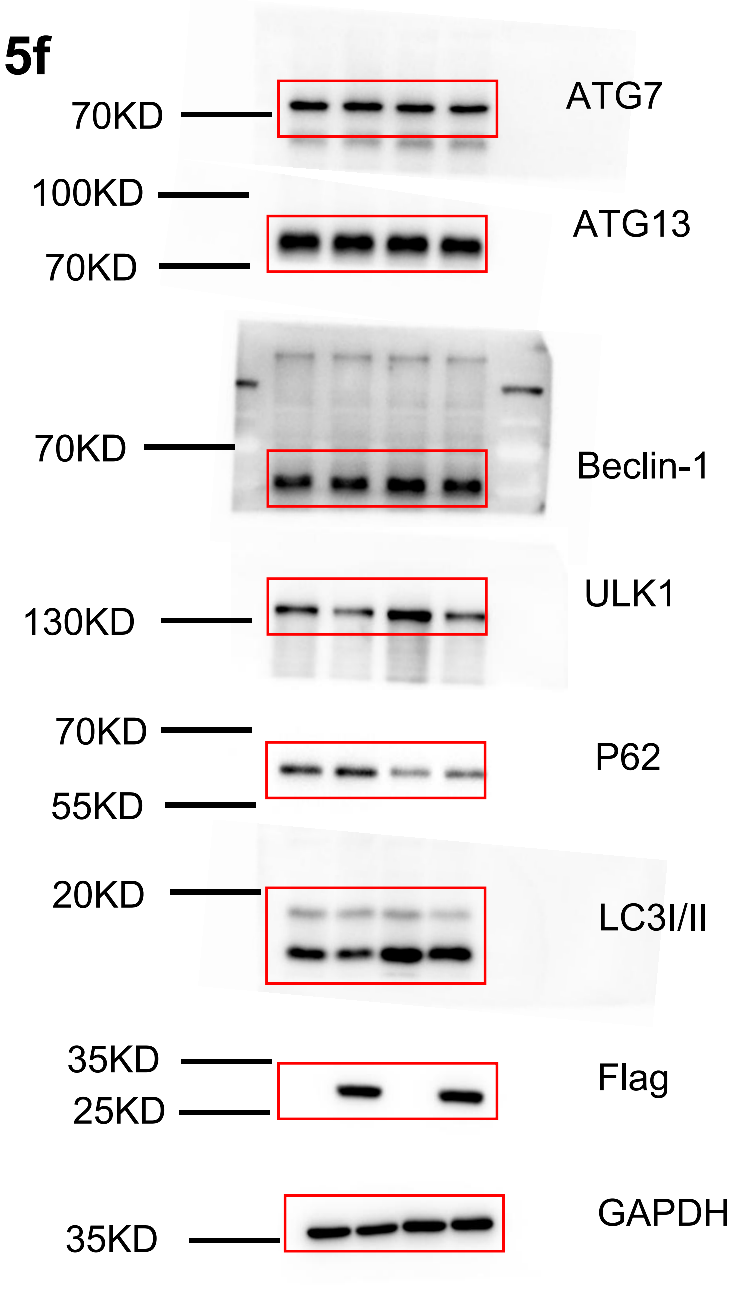
ATG7
70KD
100KD
ATG13
70KD
70KD
Beclin-1
ULK1
130KD
70KD
P62
55KD
20KD
LC3I/II
35KD
Flag
25KD
GAPDH
35KD

Fig. 6b

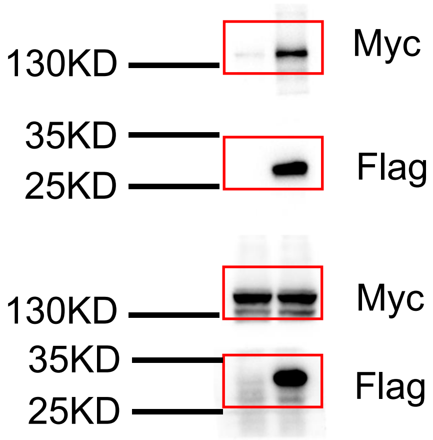

Fig. 6c

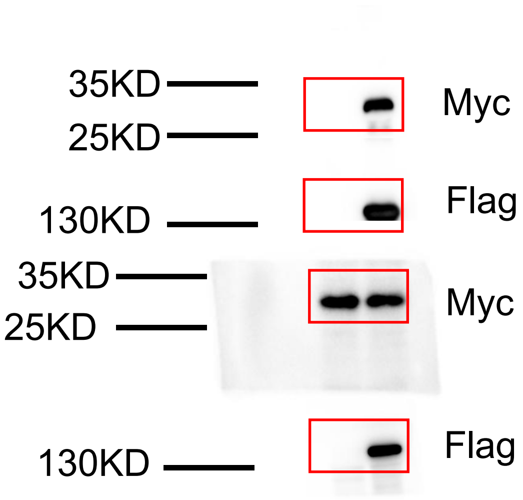

Fig. 6d

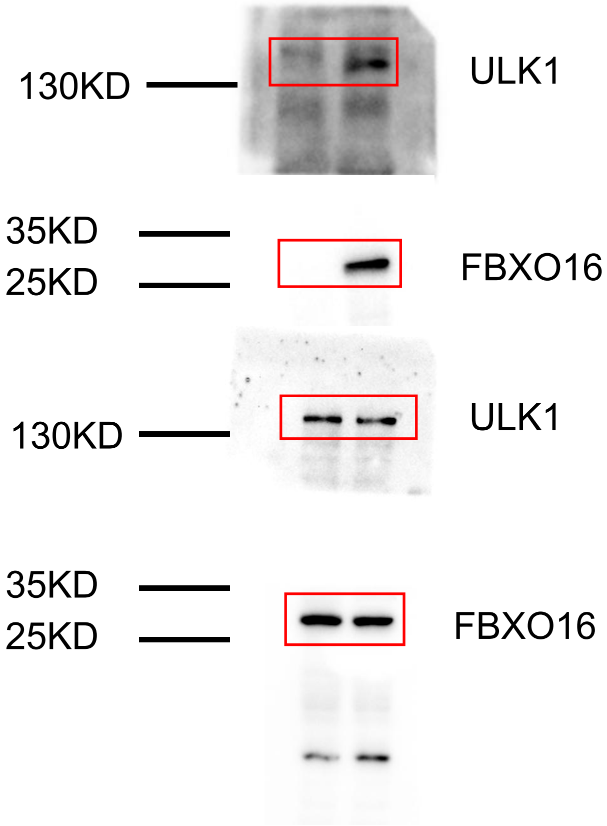

Fig. 6e

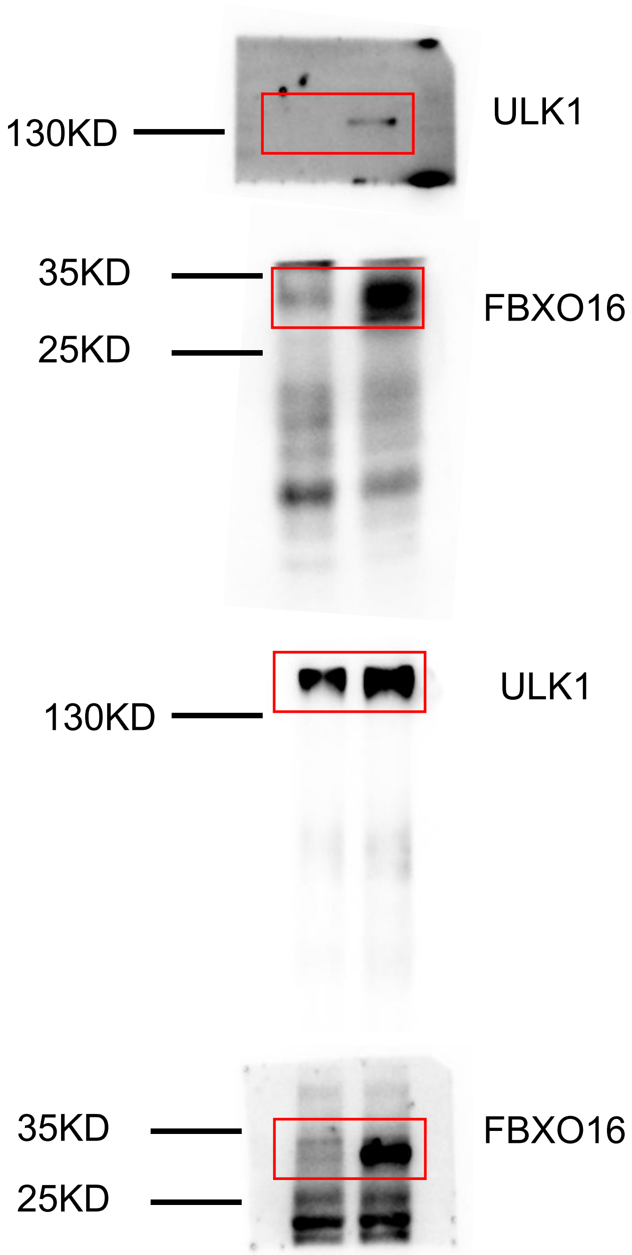

Fig. 6f

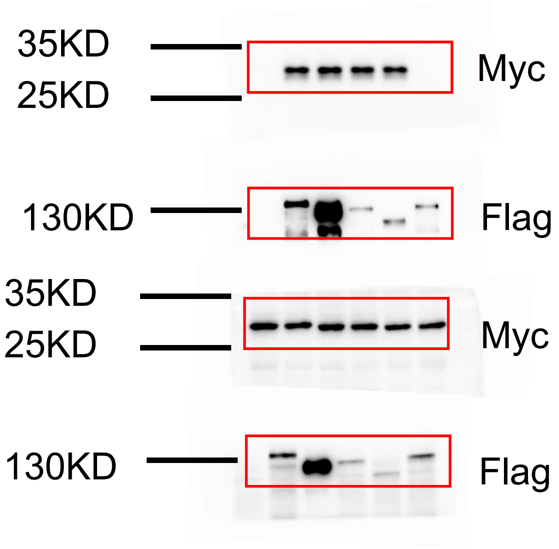

Fig. 6g

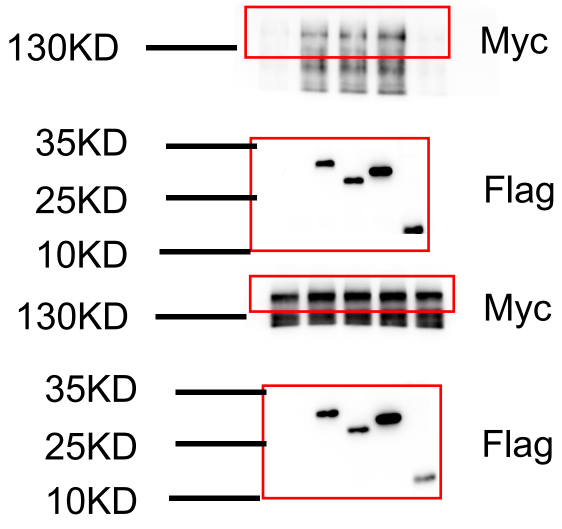

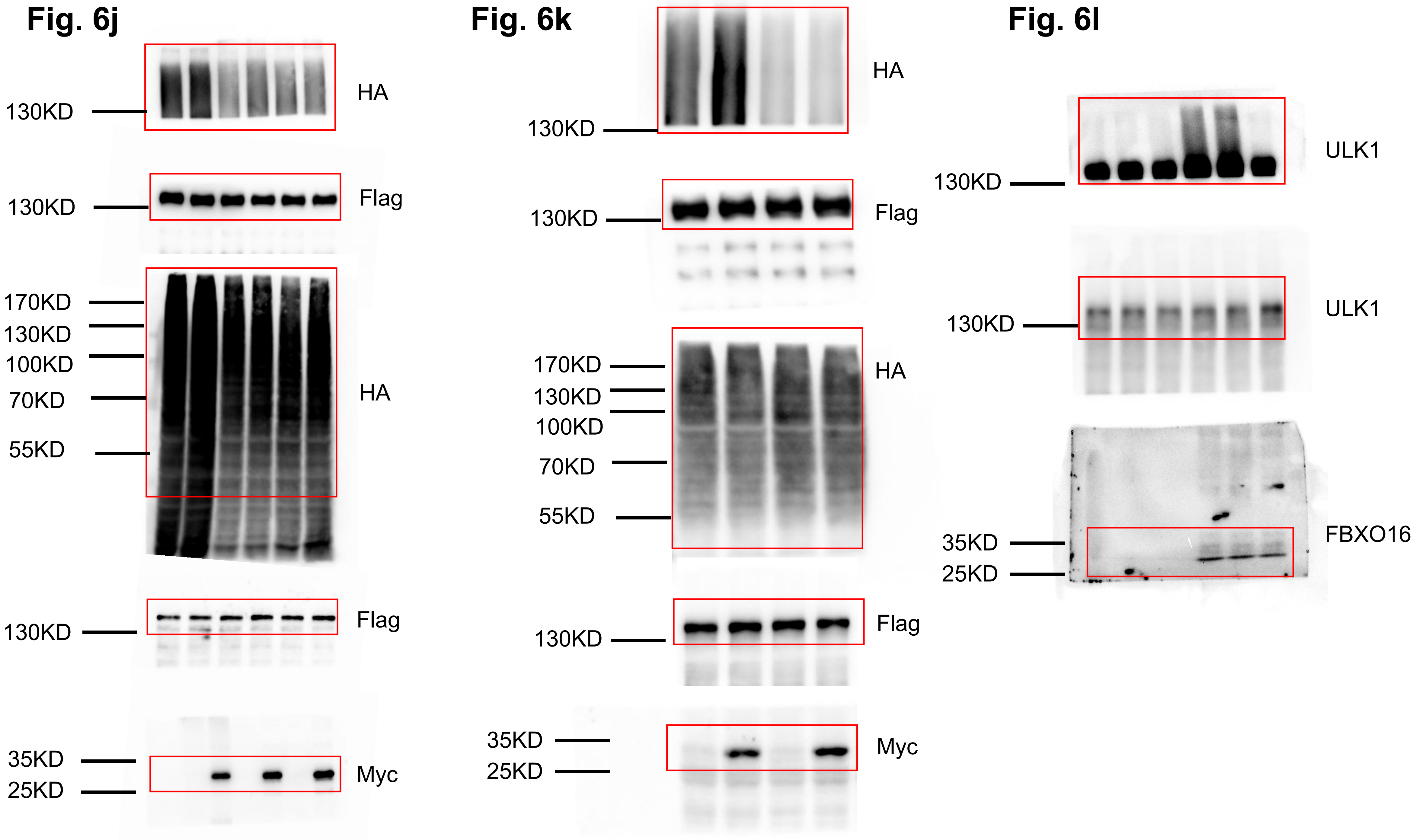
Fig. 6j
HA
130KD
Flag
130KD
170KD
130KD
100KD
70KD
55KD
HA
Flag
130KD
Myc
35KD
25KD
Fig. 6k
HA
130KD
Flag
130KD
170KD
130KD
100KD
70KD
55KD
HA
Flag
130KD
Myc
35KD
25KD
Fig. 6l
ULK1
130KD
ULK1
130KD
FBXO16
35KD
25KD

Fig. 7a Upper panel

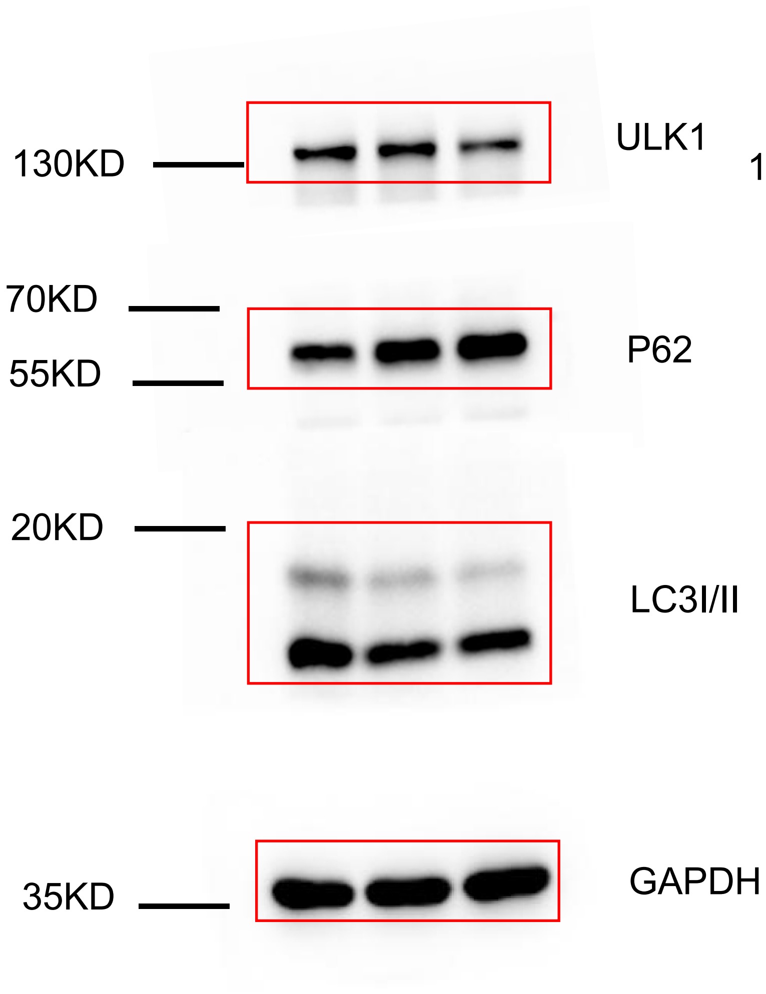

Fig. 7a Lower panel

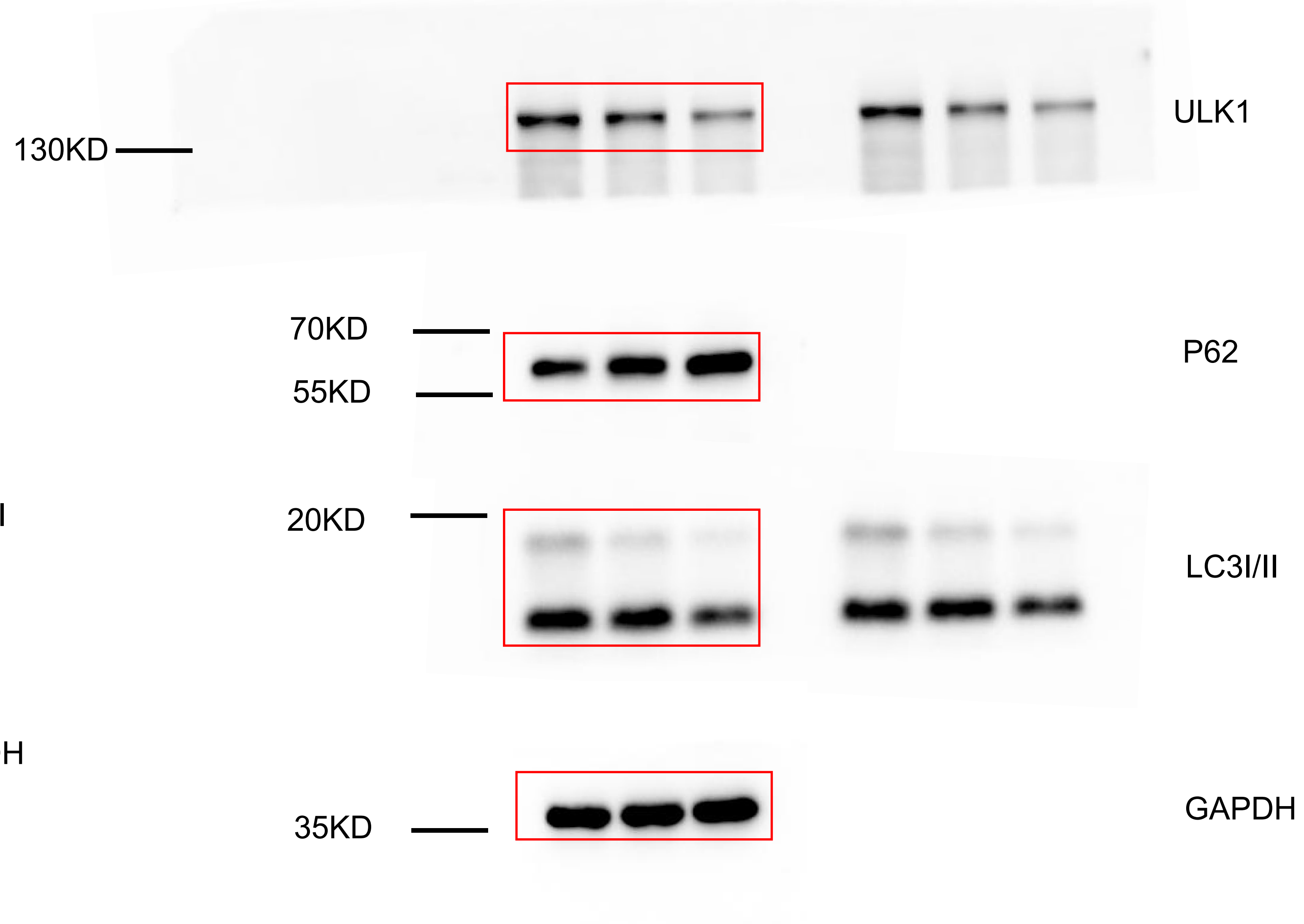

Fig. 7b

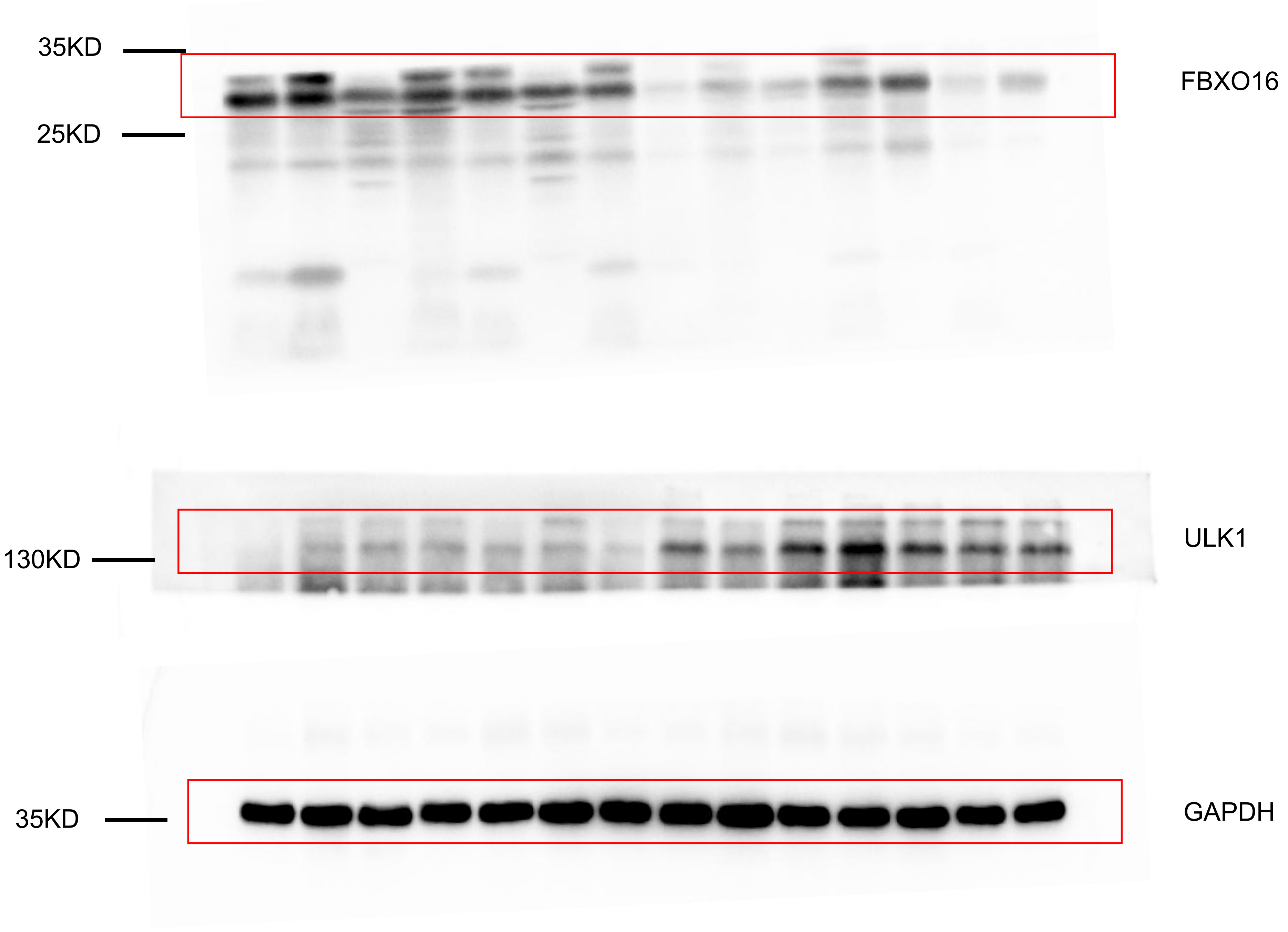

Fig. S4b

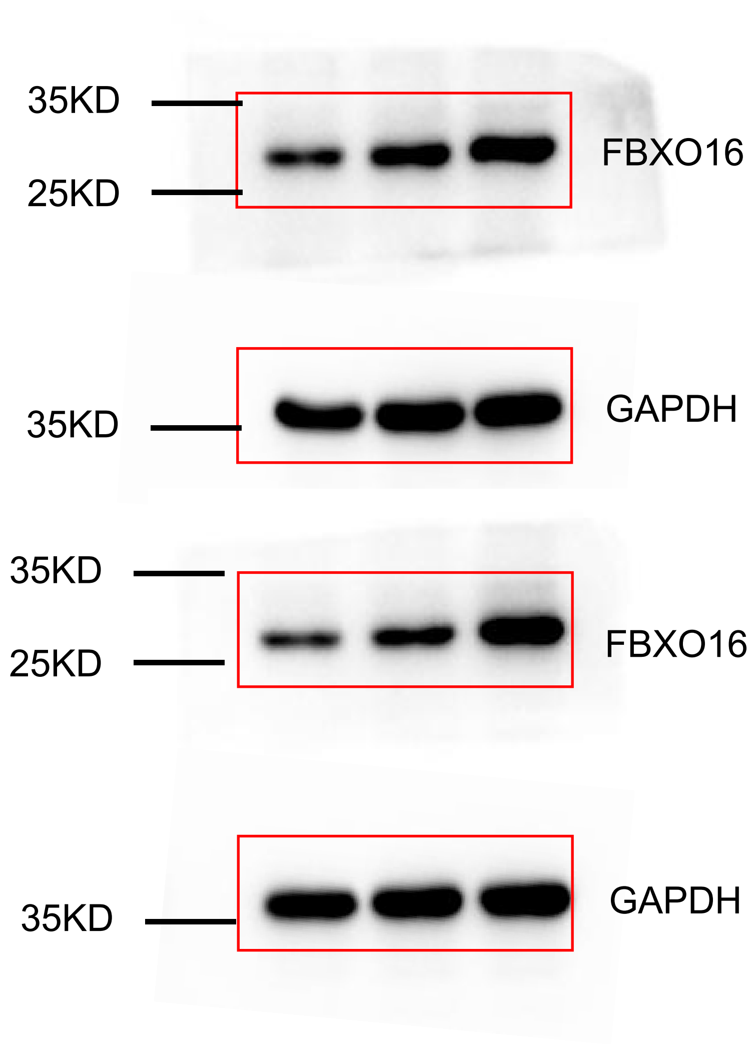

Fig. S4c

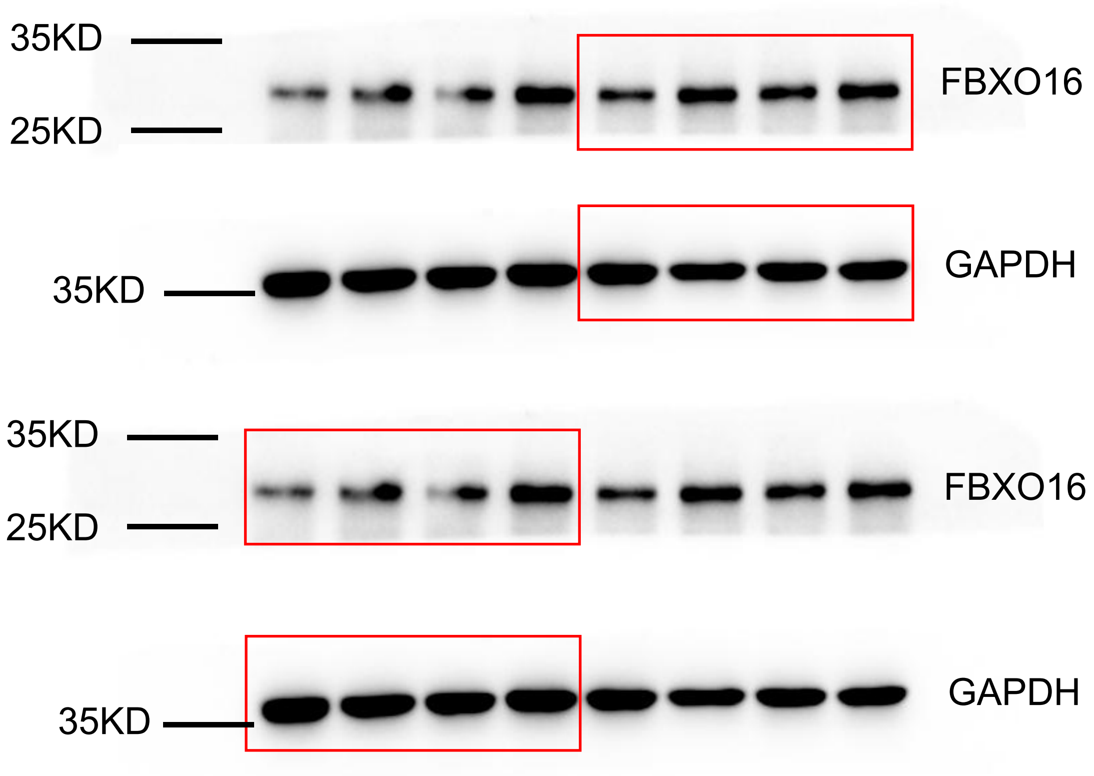

Fig. S4d

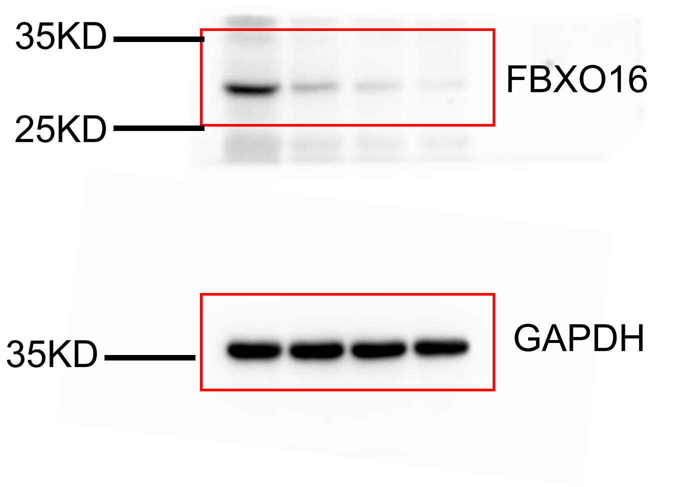

Fig. S4f

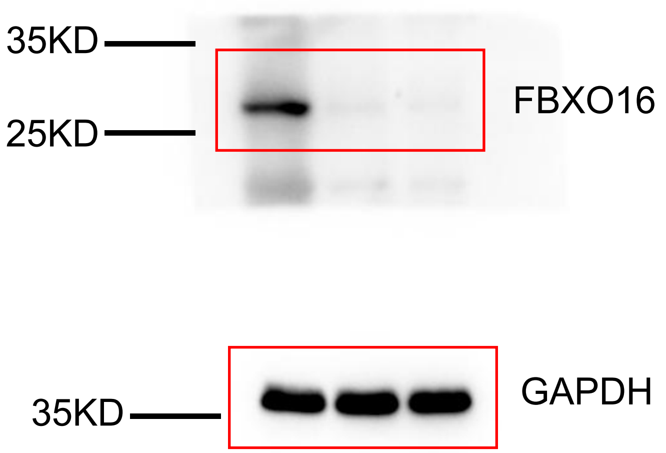

Fig. S5b

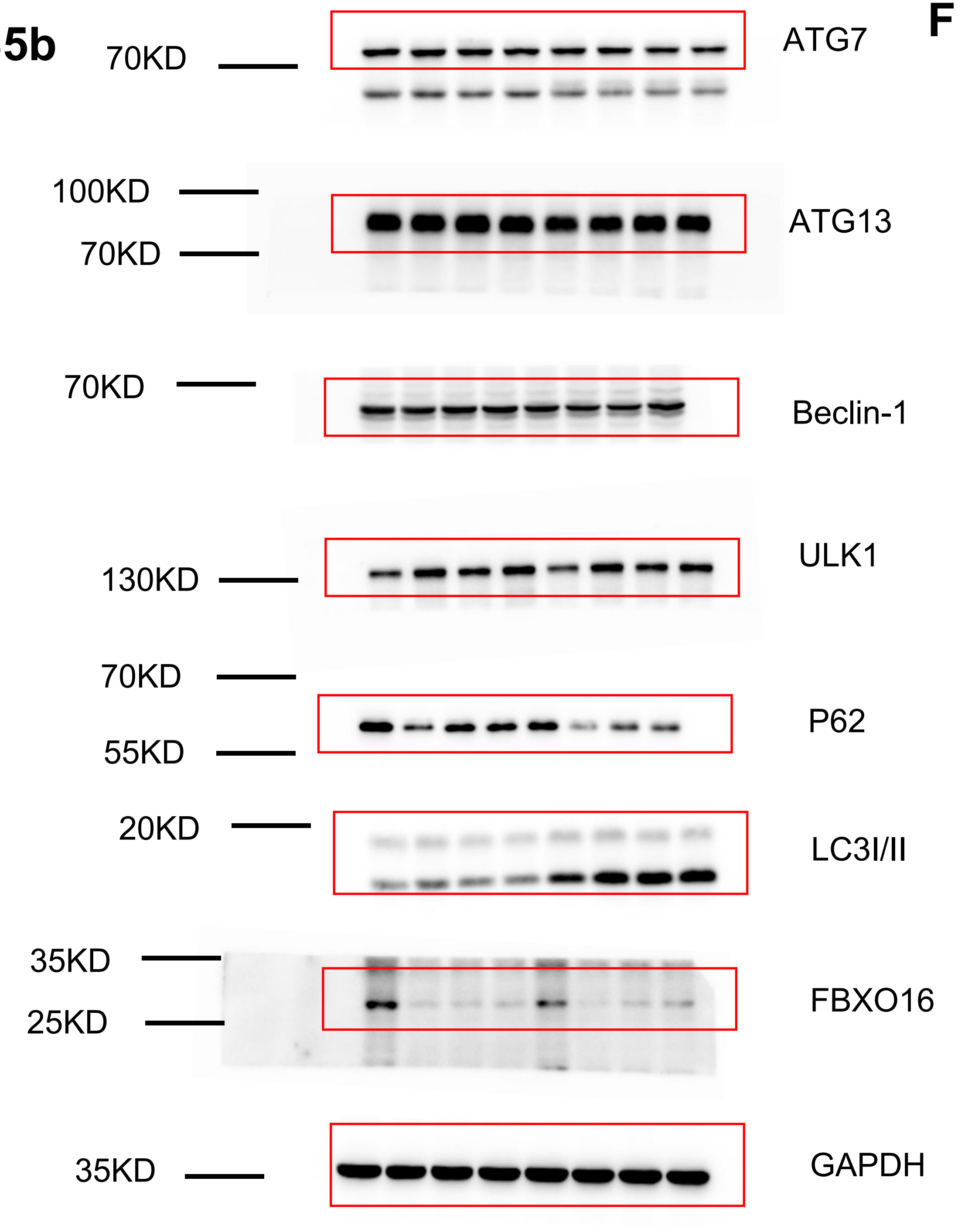

Fig. S5c

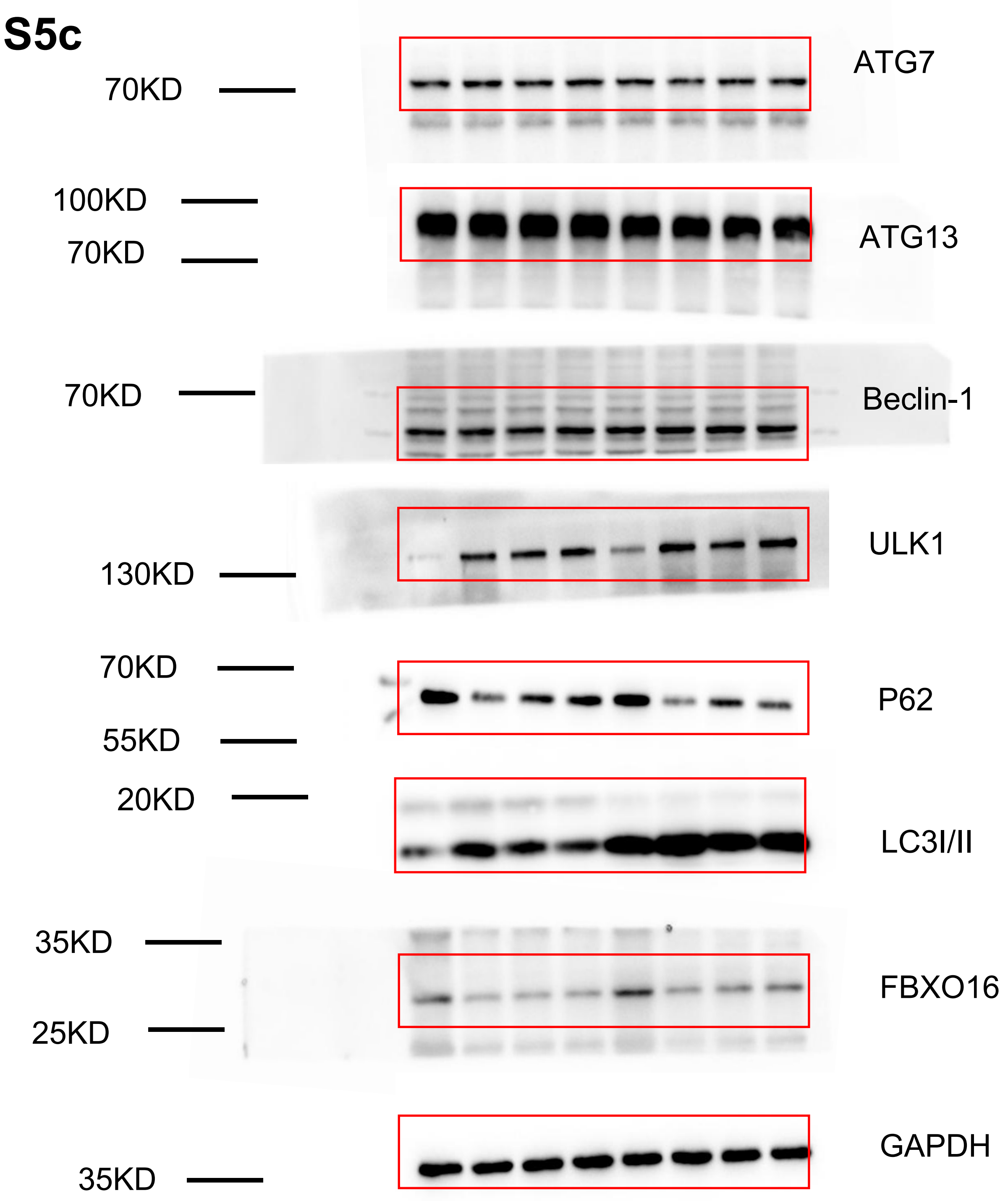

Fig. S5d

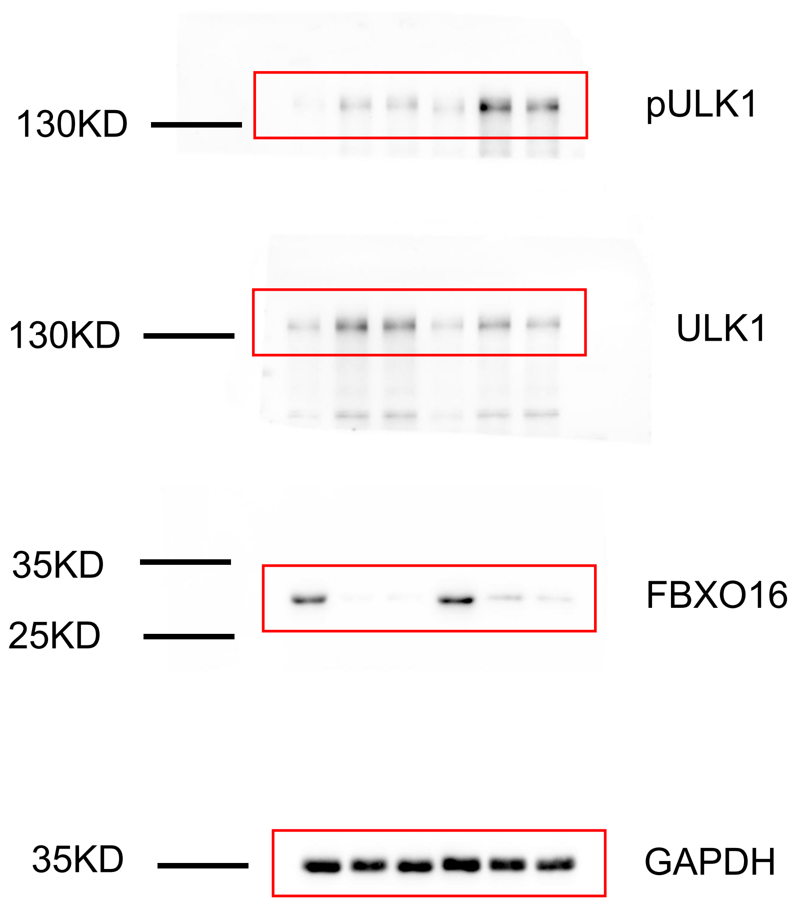

Fig. S5e

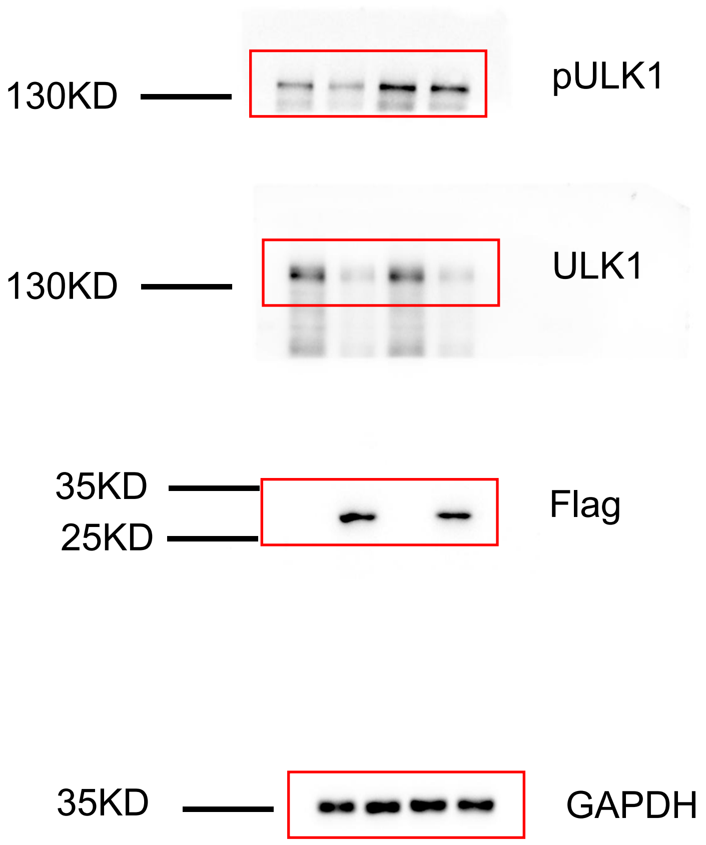

Fig. S6a

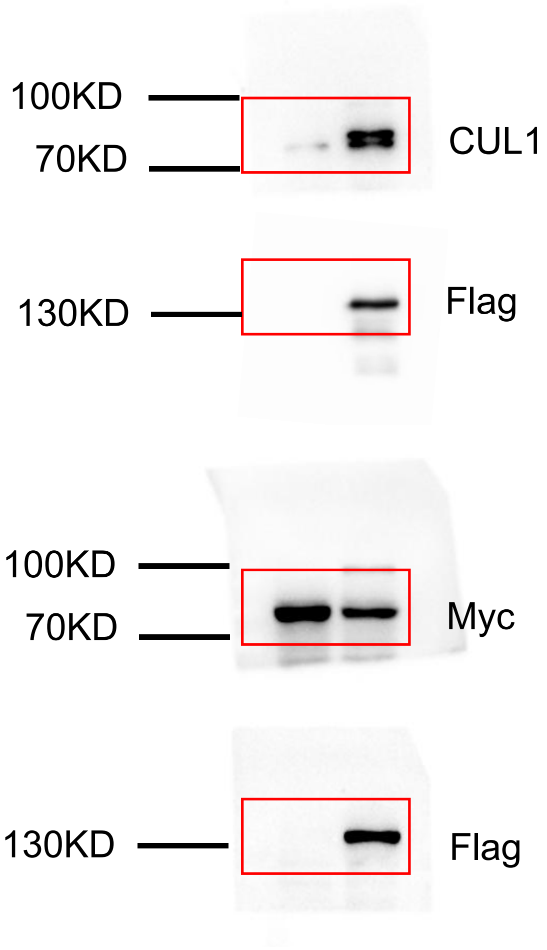

Fig. S6b

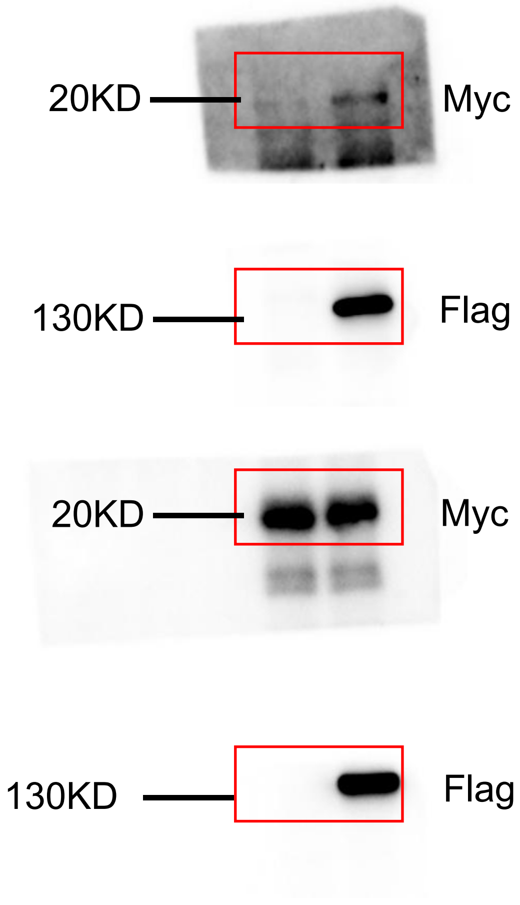

Fig. S6c

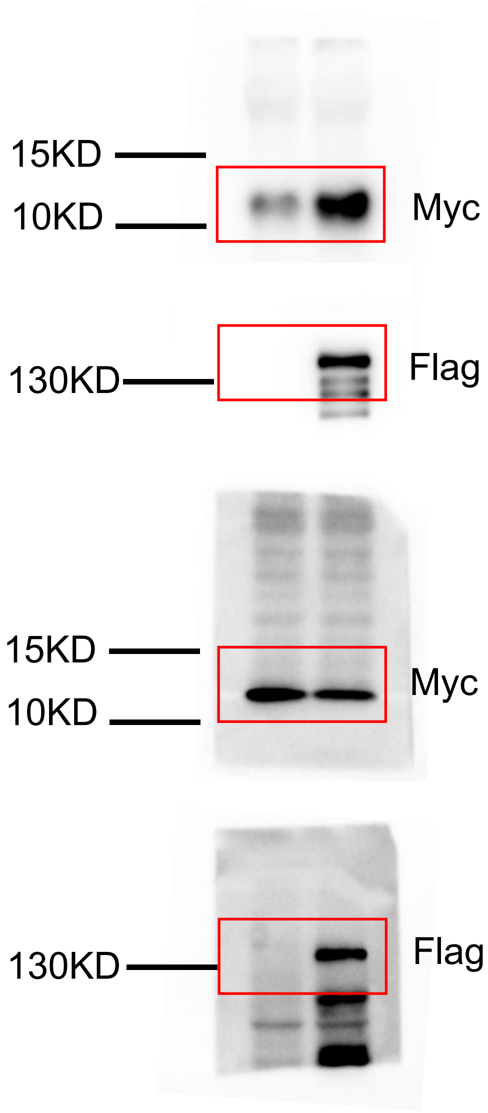

Fig. S6d

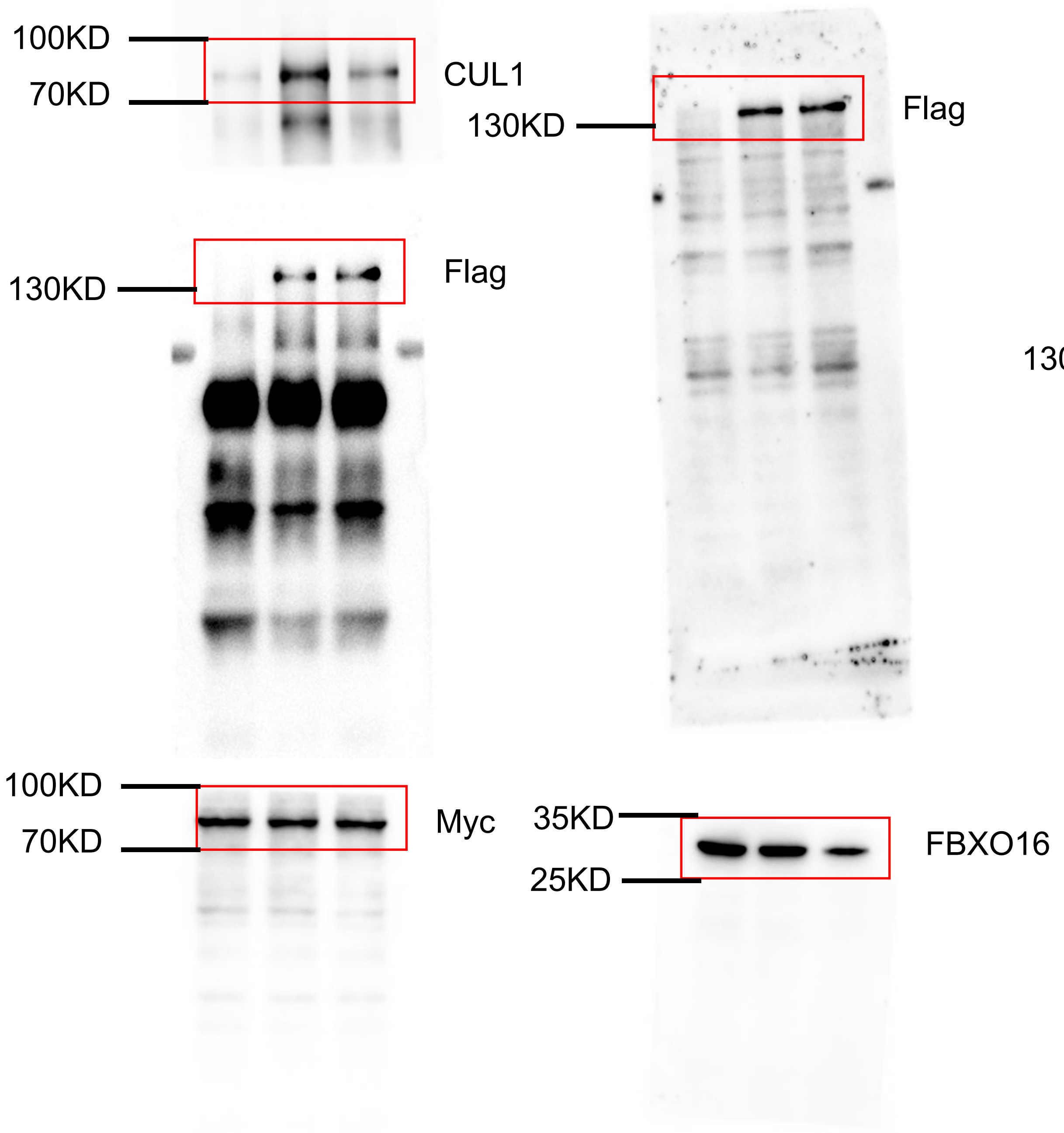

Fig. S6e

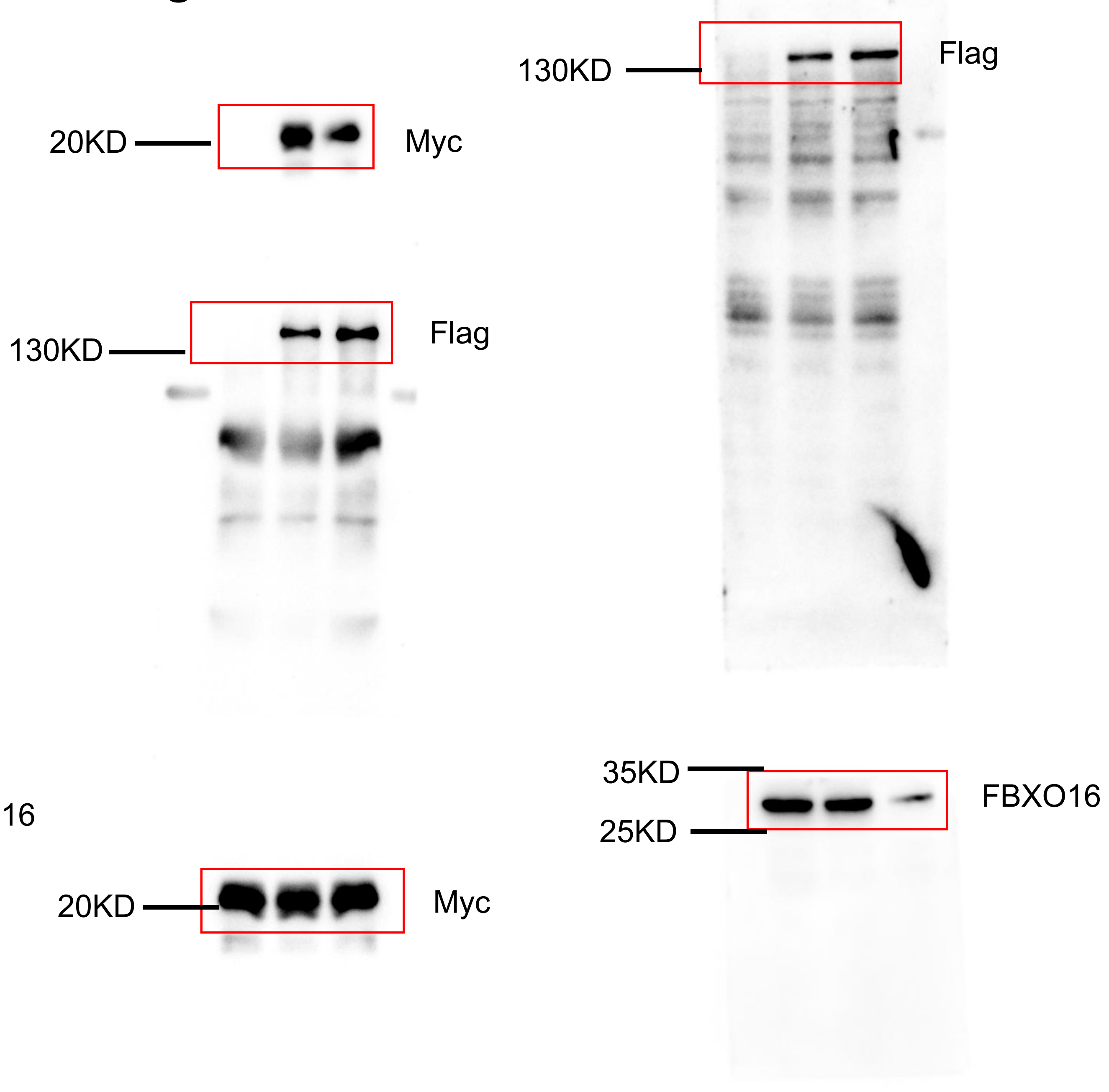

Fig. S6f

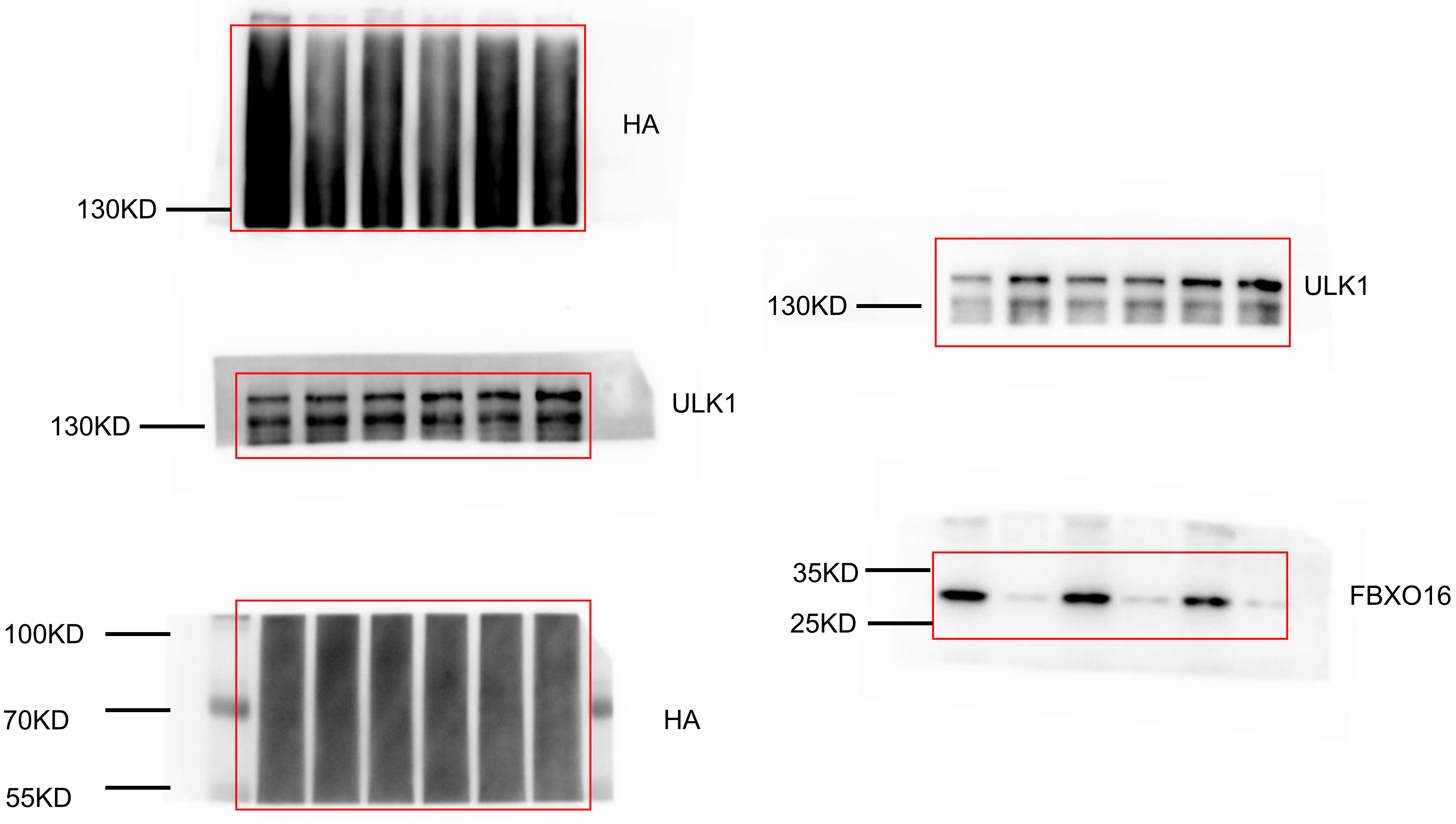
HA
130KD
ULK1
130KD
100KD
70KD
55KD
HA
130KD
ULK1
35KD
25KD
FBXO16

Fig. S6g

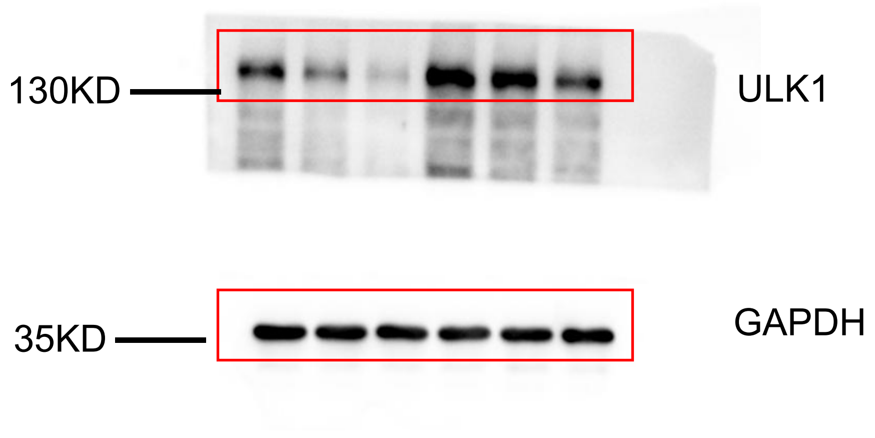

Fig. S6i

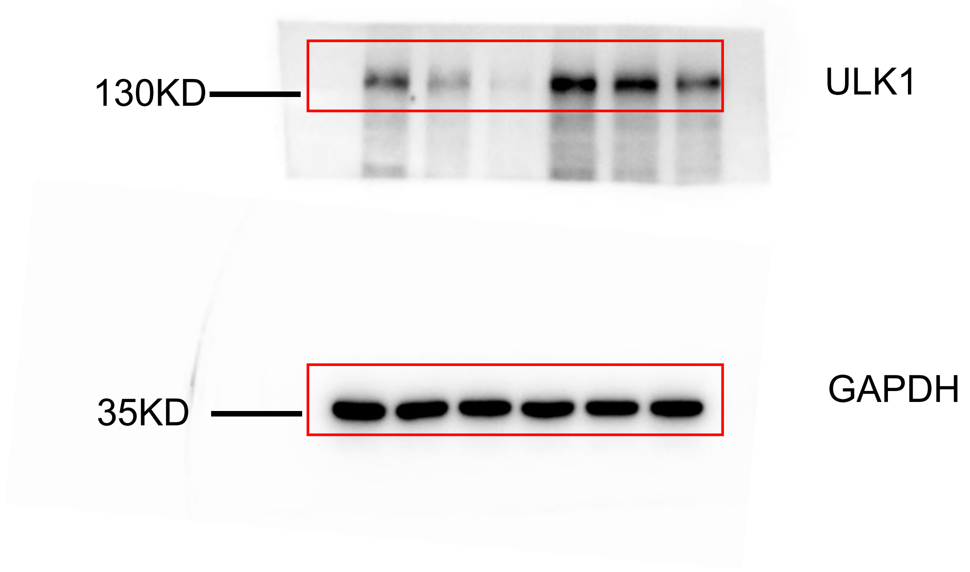

Fig. S7a

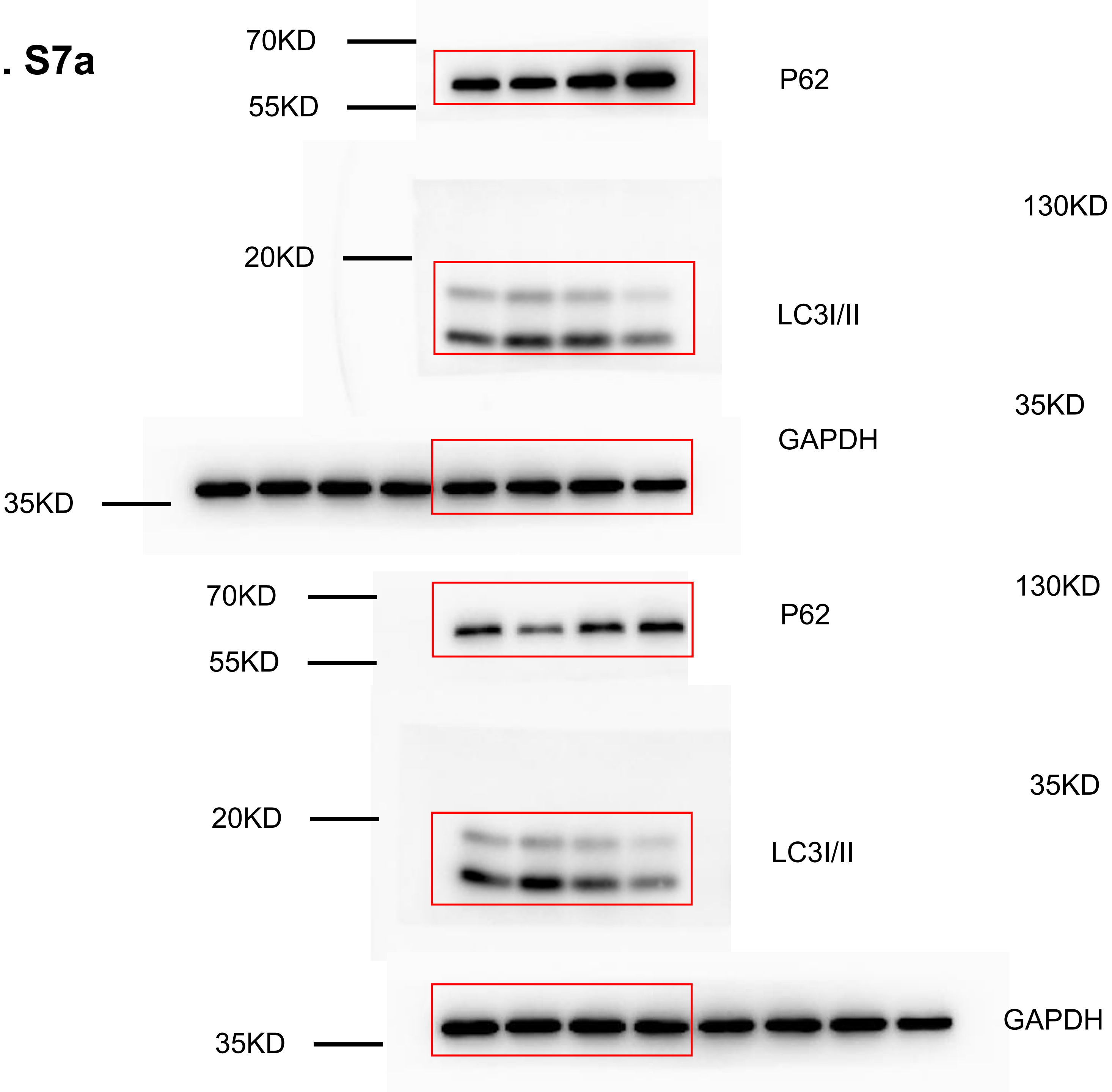

Fig. S7b

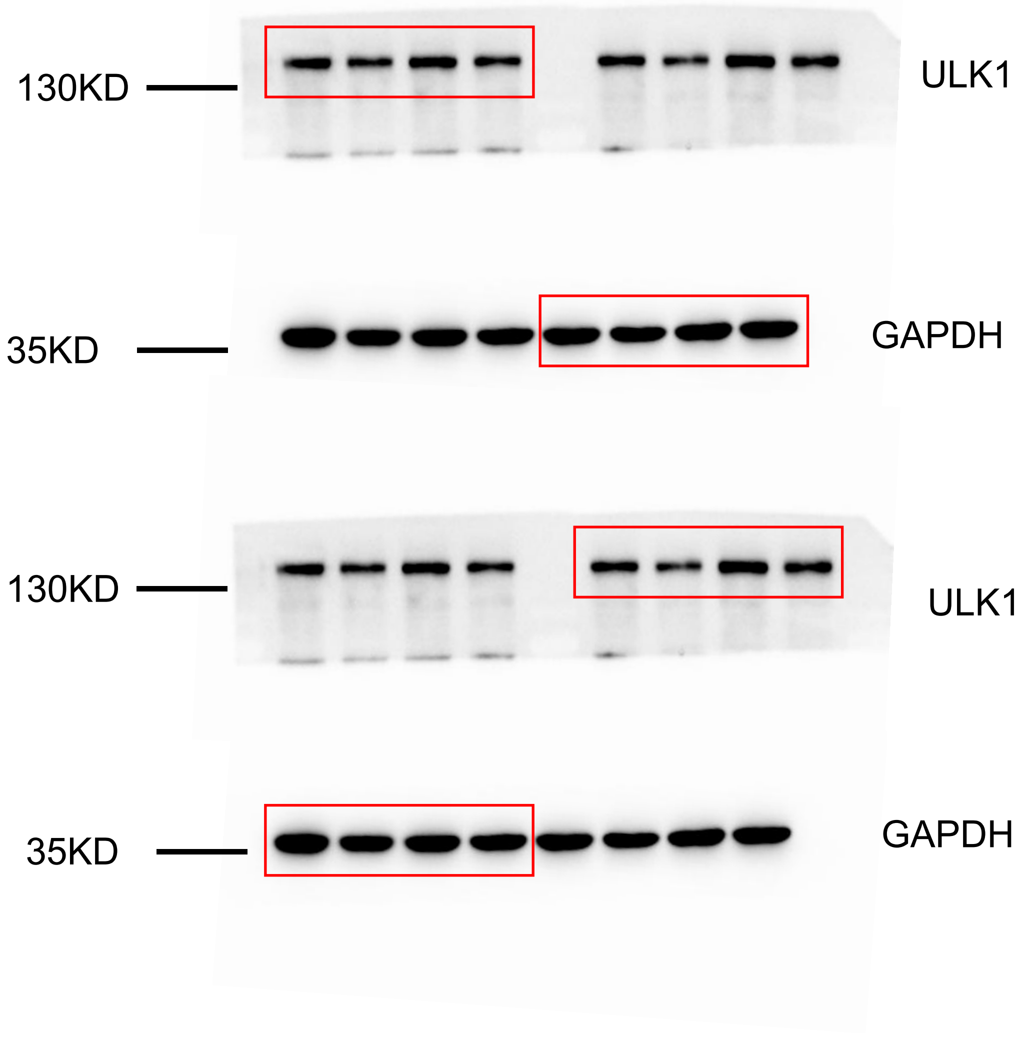

Fig. S7c Upper panel

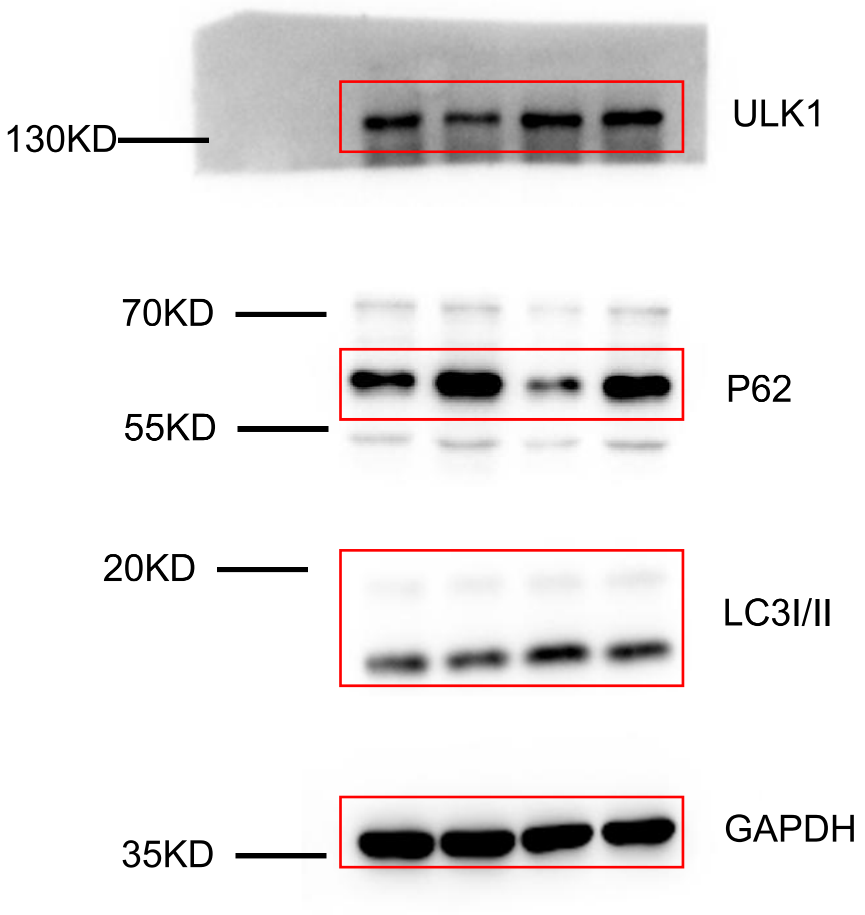

Fig. S7c Lower panel

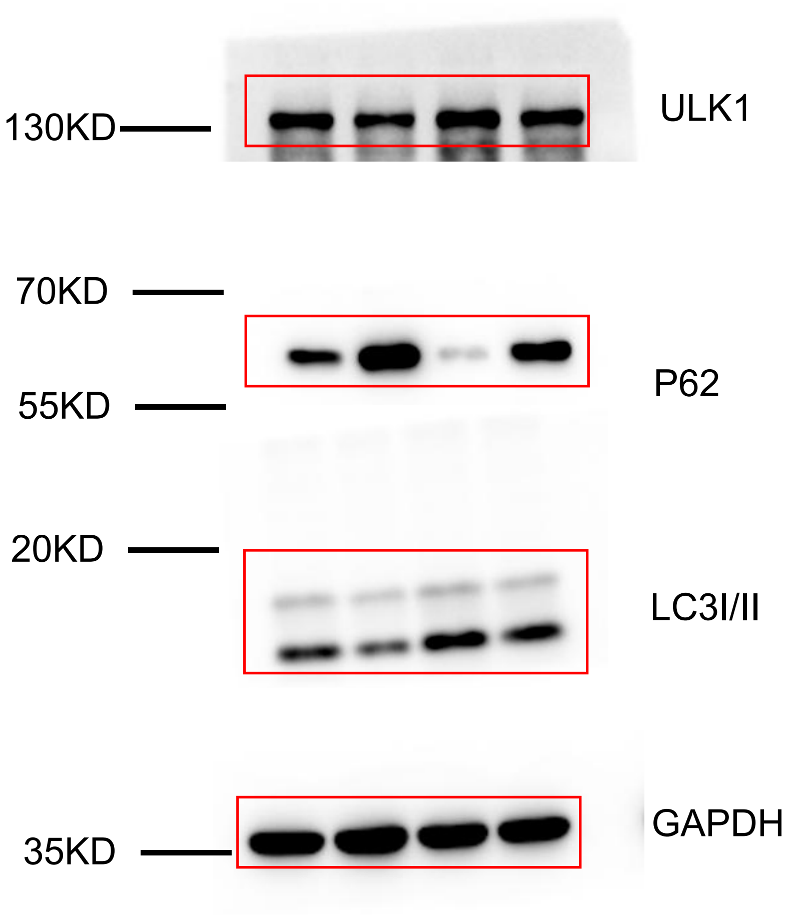

Supplement: Supplementary file 2 — Uncropped WB [file 41419_2024_7120_MOESM2_ESM.pdf]
